# Lung-gut axis of microbiome alterations following co-exposure to ultrafine carbon black and ozone

**DOI:** 10.1186/s12989-023-00528-8

**Published:** 2023-04-21

**Authors:** Md Habibul Hasan Mazumder, Jasleen Gandhi, Nairrita Majumder, Lei Wang, Robert Ian Cumming, Sydney Stradtman, Murugesan Velayutham, Quincy A. Hathaway, Jonathan Shannahan, Gangqing Hu, Timothy R. Nurkiewicz, Robert M. Tighe, Eric E. Kelley, Salik Hussain

**Affiliations:** 1 Department of Physiology, Pharmacology, and Toxicology, Center for Inhalation Toxicology (iTOX), School of Medicine, West Virginia University, Morgantown, WV 26506 USA; 2 Center for Inhalation Toxicology (iTOX), School of Medicine, West Virginia University, Morgantown, WV 26506 USA; 3 Department of Microbiology, School of Medicine, West Virginia University, Morgantown, WV 26506 USA; 4 Department of Medicine, Duke University Medical Center, Durham, NC 2927 USA; 5 School of Health Sciences, Purdue University, West Lafayette, IN 47907 USA; 6 Department of Biochemistry and Molecular Medicine, School of Medicine, West Virginia University, Morgantown, WV USA; 7 Heart and Vascular Institute, School of Medicine, West Virginia University, Morgantown, WV USA

**Keywords:** Ozone, Ultrafine carbon black, Microbial dysbiosis, Inhalation, Co-exposure, Inflammation, EPR

## Abstract

**Background:**

Microbial dysbiosis is a potential mediator of air pollution-induced adverse outcomes. However, a systemic comparison of the lung and gut microbiome alterations and lung-gut axis following air pollution exposure is scant. In this study, we exposed male C57BL/6J mice to inhaled air, CB (10 mg/m^3^), O_3_ (2 ppm) or CB + O_3_ mixture for 3 h/day for either one day or four consecutive days and were euthanized 24 h post last exposure. The lung and gut microbiome were quantified by 16 s sequencing.

**Results:**

Multiple CB + O_3_ exposures induced an increase in the lung inflammatory cells (neutrophils, eosinophils and B lymphocytes), reduced absolute bacterial load in the lungs and increased load in the gut. CB + O_3_ exposure was more potent as it decreased lung microbiome alpha diversity just after a single exposure. CB + O_3_ co-exposure uniquely increased *Clostridiaceae* and *Prevotellaceae* in the lungs. Serum short chain fatty acids (SCFA) (acetate and propionate) were increased significantly only after CB + O_3_ co-exposure. A significant increase in SCFA producing bacterial families (*Ruminococcaceae*, *Lachnospiraceae*, and *Eubacterium*) were also observed in the gut after multiple exposures. Co-exposure induced significant alterations in the gut derived metabolite receptors/mediator (*Gcg, Glp-1r, Cck*) mRNA expression. Oxidative stress related mRNA expression in lungs, and oxidant levels in the BALF, serum and gut significantly increased after CB + O_3_ exposures.

**Conclusion:**

Our study confirms distinct gut and lung microbiome alterations after CB + O_3_ inhalation co-exposure and indicate a potential homeostatic shift in the gut microbiome to counter deleterious impacts of environmental exposures on metabolic system.

**Supplementary Information:**

The online version contains supplementary material available at 10.1186/s12989-023-00528-8.

## Introduction

Air pollution is among the most severe environmental health threats [1]. Air pollution exposure accounts for more than 6.5 million deaths per year. Approximately 134 million individuals in the US were exposed to unhealthy levels of particulate matter (PM) during 2020 [2]. The toxicity of the individual components of air pollution (PM, O_3_, NOx- Nitrogen oxides) are well established and form the basis of current regulatory decision making. However, interactive outcomes between different components of air pollution are not well established [3]. We previously reported that ozone (O_3_) and carbon black (CB) inhalation co-exposure induces significantly greater lung injury and inflammation as well as macrophage driven endothelial cell damage [4]. In our studies, we used CB as a surrogate for the ultrafine PM carbon core. Ultrafine CB can induce significant genotoxicity, pulmonary toxicity, and developmental toxicity through direct or indirect mechanisms [4]. Alternatively, O_3_ is a major component of air pollution which is produced by the interaction between ultraviolet light with different pollutants, such as volatile organic compounds, and nitrogen and sulfur oxides [5, 6]. O_3_ induces pulmonary inflammation in humans and animal models [7, 8]. Despite clear associations between independent O_3_ and PM exposures and pre-existing respiratory conditions such as COPD, and asthma [9, 10], recent epidemiological studies suggest potential synergy and interactive outcomes [11]. Previous PM and O_3_ co-exposures studies were mainly focused on cardiovascular outcomes and only few focused on respiratory outcomes [12–20].

Microbiome is defined as the collection of genetic material co-existing in a specific environment [21]. The microbiome modulates multiple physiologically important responses that include polysaccharide digestion, nutrient production, pathogen evasion, detoxification, and immune regulation [22–24]. The respiratory microbiome is dynamic in nature and consists of the nasopharyngeal airway, upper respiratory tract and lower respiratory tract microbiome. The composition of the respiratory tract microbiome is influenced by various factors, e.g., genetics, environmental exposures, immune status, and prior infections [25, 26]. Altered respiratory tract microbiota composition is associated with multiple respiratory pathologies such as chronic obstructive pulmonary disease (COPD), asthma, idiopathic pulmonary fibrosis (IPF), and cystic fibrosis (CF) [27–29]. Only a handful of studies have examined microbiome dynamics in air pollution exposure induced pulmonary responses [30–33]. Similarly, the impact of air pollution exposure on the gut microbiota is only recently gaining attention and the potential for bidirectional connection exist between the lungs and gut microbiome [34]. The majority of published evidence focuses on either the lung or gut microbiome and only a handful of studies have defined microbiome changes in the lung and gut in the same animals [35–37]. Exposure to diesel exhaust particles (DEP) induces systemic inflammation and alteration of gut *Actinobacteria*, *Proteobacteria,* and *Verrucomicrobia* [38]. Moreover, prominent microbial changes, alteration in host-derived metabolites, and gut permeability were observed in ApoE knockout mice in response to PM exposure [39]. PM exposure induced microbial dysbiosis, exacerbation of respiratory dysfunction, acute respiratory distress syndrome, and inflammatory bowel disease [40–42]. However, these studies are performed using individual particulates or gaseous components of air pollution and the impacts of a realistic particle and gas mixture inhalation model are still not reported. Analysis of lung and gut microbiome in a single organism enables to establish the lung-gut axis with more accuracy. It also enables to reduce multiple confounding/artifacts such as housing and handling/stress that can impact microbiome outcomes [43–45]. This also results in reducing the impact of inter individual/animal variability and thus result in more robust data.

We recently reported that repeated inhalation exposure to ultrafine CB and O_3_ caused pulmonary injury and progressive mitochondrial dysfunction [3]. The present study was designed to define changes in the lung and gut microbiome after single or repeated inhalation exposure in male C57BL/6J mice. We hypothesized that inhalation co-exposure to ultrafine CB and O_3_ will induce unique microbiome alterations in the lung and gut. We also sought to determine changes that are uniquely induced by the co-exposure to define impacts of co-exposure. In addition, we defined differences in serum SCFA contents and oxidant/antioxidant balance as potential systemic mediators of microbiome-induced pulmonary and systemic alterations.

## Materials and methods

### Exposure system and aerosol characterization

A whole-body inhalation exposure system was designed to expose animals to aerosols of carbon black (CB), ozone (O_3_), or a mixture of CB + O_3_. The exposure system has been detailed previously [4]. Briefly, bulk CB (Printex 90, provided as a gift from Evonik, Germany) was aerosolized with a high-pressure acoustical generator (HPAG, IEStechno, Morgantown, WV). The output from the HPAG was further de-agglomerated with a Venturi pump (JS-60M, Vaccon, Medway MA). The real time particle number concentration (mg/m^3^) was monitored using a light scattering device (DataRAM, pDr-1500, Thermo Environmental Instruments Inc, Franklin, MA). O_3_ was generated by passing pure oxygen through a corona discharged O_3_ generator (HTU500AC, Ozone Solutions, Hull, IA). The O_3_ level was monitored in real-time with a calibrated O_3_ monitor (Model 202, 2B Technologies, Inc., Boulder, CO). During co-exposure, the generated O_3_ was mixed with CB and introduced into the 150 L stainless steel exposure chamber (Cube 150, IEStechno, Morgantown, WV) housing up-to 36 mice in individual stainless steel mesh cages. CB and O_3_ levels were monitored and maintained at predefined levels using automated feed-back loops. The temperature (20–22 °C) and humidity (50–70%) were monitored and maintained throughout the exposure. The particle size distribution was measured from the exposure chamber using the (1) Electrical Low-Pressure Impactor (ELPI+, Dakati, Tempera, Finland), (2) Aerosol Particle Sizer (APS 3321, TSI Inc Shoreview, MN), 3) Scanning mobility particle sizer (SMPS 3938, TSI Inc. Shoreview, MN), and 4) Nano Micro-orifice Uniform Deposit Impactor (Moudi 115R, MSP Corp, Shoreview, MN). The morphology of the aerosolized particles was observed using a field-emission scanning electron microscope (Hitachi S4800, Tokyo, Japan) and transmission electron microcopy (JEOL JEM-2100 TEM) as described previously [3].

### Murine model

**Fig. 1:**
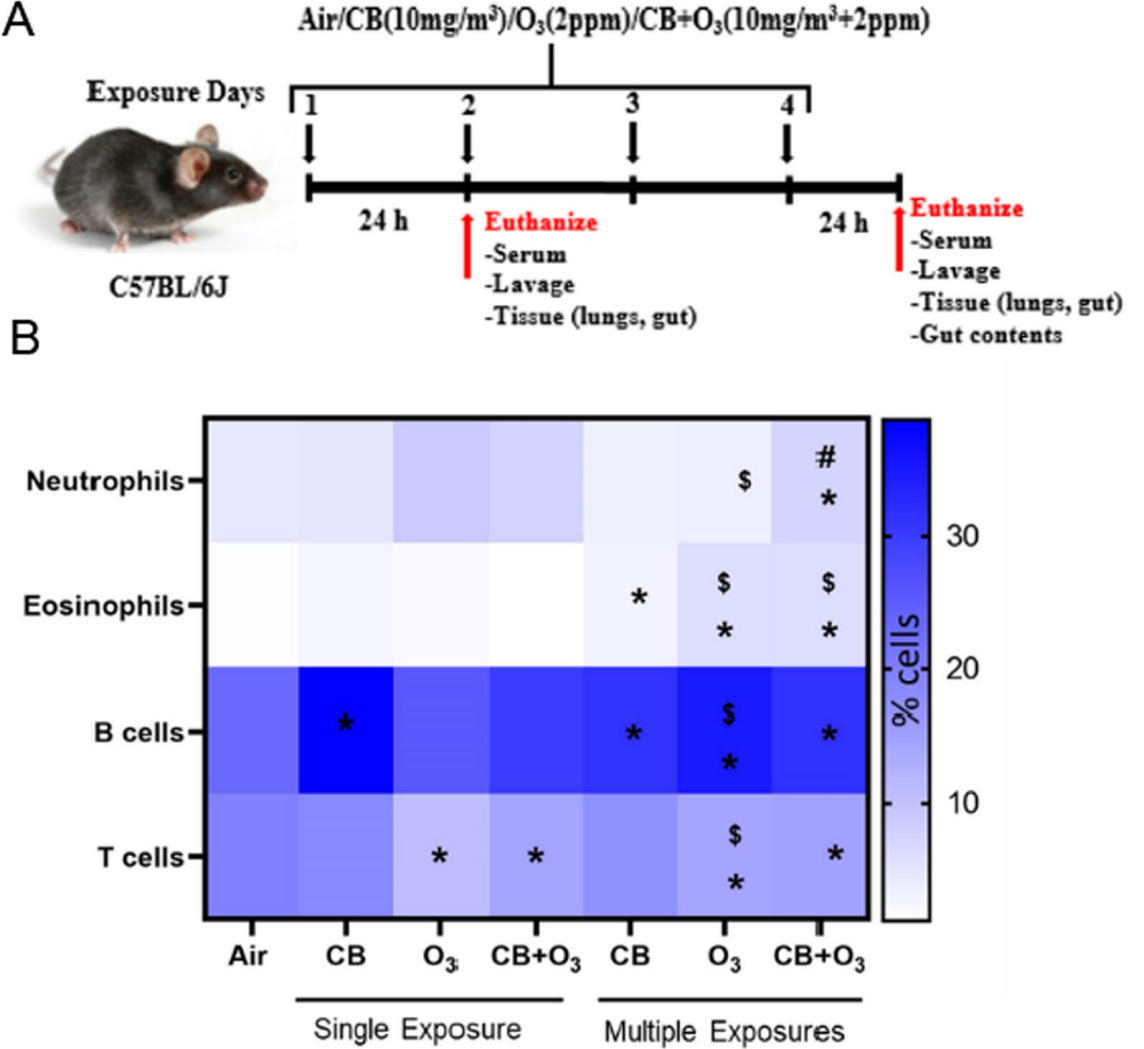
Study layout and analysis of lung inflammation. **A** Layout of animal exposure experiments. **B** Heat-map showing alterations in immune cell profile. C57BL/6J mice (8–12 weeks) were exposed to air, CB (10 mg/m^3^), O_3_ (2 ppm) or CB + O_3_ for 3 h for either one or four exposures and euthanized 24 h post last exposure. Data are presented as mean ± standard error of mean (SEM) and analyzed by Two-way Analysis of Variance (ANOVA) followed by Tukey’s post hoc test. Lung tissues were homogenized, stained and analyzed in LSRFortessa II. n = 3–5. *p ≤ 0.05. *denotes significantly difference from air, # denotes significantly different from O_3_ at the same time point, and $ denotes significantly different between single and multiple exposure groups

All animal studies were approved by the West Virginia University (WVU) Animal Care and Use Committee. C57BL/6J male (8–10 weeks old) mice were purchased from Jackson Laboratory (Bar Harbor, ME) and acclimated at the WVU animal care facility for 7 days before exposure. Mice were kept in HEPA filtered cages, provided with chow and water ad libitum and maintained at a 12-h light/dark cycle. Animals were maintained in social housing (five mice/cage). Mice were single housed during exposure (3 h per exposure) in steel mesh cages. The temperature (20–22 °C) and humidity (50–70%) were monitored and maintained throughout the exposure. Mice were exposed Monday-Thursday starting at 7:00 AM each day. Mice were provided with chow and water ad libitum and maintained at a 12-h light/dark cycle. Mice were randomly divided into four groups: (1) control/filtered air, (2) CB (10 mg/m^3^), (3) O_3_ (2 ppm), and 4) CB + O_3_ (10 mg/m^3^ + 2 ppm) co-exposure. Exposures were performed in the WVU iTOX Inhalation Facility. Animals were exposed for 3 h a day, once or daily up to four days and weights were monitored daily and reported in Additional file 1: Figure S1. Mice were euthanized by intraperitoneal injection of Fatal Plus (250 mg/kg) 24 h after the final inhalation exposure. Schematics for exposure is presented in Fig. 1A.

### Bronchoalveolar lavage analysis

Following euthanasia, approximately, 1 mL ice cold sterile PBS was instilled through the trachea into the lungs via a syringe three times to obtain approximately 3 mL broncho-alveolar lavage fluid (BALF). BALF was centrifuged at 600 RPM for 5 min at 4 °C, BALF supernatant was collected, and stored at −80 °C for further experiments.

### Flow cytometry

Following euthanasia, freshly collected lung tissue was used for flow cytometry analysis. Briefly, the lung was inflated and collected in a digestion buffer composed of DMEM (Dulbecco’s Modified Eagle Medium) F-12 (Gibco) containing DNAseI (0.33 U/mL, Sigma Aldrich), dispase (5 U/mL, Gibco), and collagenase I (450 U/ mL, Roche) [46]. Lungs were cut and incubated in 37 °C for 20 min in digestion media with periodic shaking. At the end of incubation period, 10% DMEM F-12 (Gibco) was added to the cells and single cell suspension was obtained by passing through a 70 μM nylon mesh. Red blood cell (RBC) lysis was performed using RBC lysis buffer (BD Biosciences) by incubating the cells for 5 min at room temperature in the dark. The single cell suspension was counted using Countess Cell Counter (Invitrogen). Approximately 2 million cells were aliquoted and stained with viability dye (FITC), incubated with FcRBlock (Miltenyl Biotec), and stained with a mixture of fluorochrome-conjugated antibodies. Data were acquired on the BD LSR Fortessa II using BD FACSDiva software. Analysis was performed through FCS Express 7 (De Novo Software, Glendale, CA). Gating strategy and inflammatory cell identification procedure and respective clones and vendors are defined in Additional file 1: Figure S2.

### DNA isolation

Genomic DNA was extracted from the lungs and colon contents (later defined as gut) after pulverizing at -80 °C using Qiagen DNeasy Blood and Tissue kit (Qiagen, Hilden, Germany) as per manufacturer’s protocol. Low biomass specimens (lungs) were collected prior to high biomass specimens (gut) for 16 s rRNA sequencing. To avoid microbial contamination, separate sets of instruments were used to collect lung and gut. The DNA concentration and purity were measured using the Nanodrop One (Thermo Fisher Scientific, Waltham, MA). Specimens were processed in a randomized order to minimize the risk of false positive pattern formation due to reagent contamination. All the reagents were obtained from sterile and molecular biology grade preparations.

### 16 s rRNA gene sequencing and DNA quantification

Microbial DNA was analyzed through targeting the V3–V4 regions of the 16S rRNA gene, which were amplified using the KAPA HiFi HotStart PCR Kit (KAPA Byosystems, Wilmington, MA, USA). The composition of the PCR reaction included: 1X KAPA HiFi Fidelity Buffer, 0.5 mM MgCl2, 0.3 mM KAPA dNTP Mix, 0.3 μM primers, 0.5 U KAPA HiFi HotStart DNA Polymerase, and 15 ng DNA template. The PCR program consisted of the following steps: initial denaturation at 95 °C for 3 min, 15 cycles at 98 °C for 20 s, 63 °C for 30 s, and 72 °C for 30 s, and 72 °C for 5 min for the final extension. The PCR products were purified using KAPA pure beads (KAPA Byosystems, Wilmington, MA, USA) using a Magnetic Stand-96 (Thermo Fisher Scientific). The PCR products were quality controlled with a 1% Agarose Gel. The Index PCR was performed using Nextera XT DNA Library Preparation Kit (Illumina) and KAPA HiFi HotStart PCR Kit as follows: 1X KAPA HiFi Fidelity Buffer, 0.5 mM MgCl2, 0.3 mM dNTP Mix (KAPA), 0.3 μM of primers, 0.5 U DNA Polymerase (KAPA HiFi HotStart), and 5 μL of Index 1 Primers and Index 2 Primers (Nextera XT) per sample. The PCR program consisted of the following steps: initial denaturation at 95 °C for 3 min, 22 cycles at 95 °C for 30 s, 56 °C for 30 s, and 72 °C for 30 s, and 72 °C for 4 min for the final extension. The procedure was performed as described in the 16S Metagenomic Sequencing Library Preparation guide. Library products were purified as PCR products. Qubit 3.0 fluorimeter (Thermo Fisher Scientific) was used to measure the final DNA concentrations. These concentrations were validated with the Agilent 2100 Bioanalyzer (Agilent Technologies, CA, USA). The purified products were diluted to a final concentration of 4 nM. The pooled purified products were denatured, and loaded for sample analysis, as per Library Preparation guide (The 16S Metagenomic Sequencing Library Preparation guide), at a final concentration of 7 pM. The 16S rRNA gene libraries were sequenced with 2 × 300 paired end reads using the Illumina MiSeq system (Illumina).

### Sequence quality assessment and bioinformatics analysis

The microbiome samples in the fastq format were processed and analyzed using the Quantitative Insights Into Microbial Ecology (QIIME2, version 2020.11) pipeline [47]. FastQC was used to inspect the quality of fastq files [48]. In the QIIME2 environment, the paired end reads (2 × 300 bp) were demultiplexed using MiSeq Reporter. Sequences were processed to classify microbial components using the DADA2 pipeline [49]. Sequences were processed in a sequential manner through dereplication, denoising, merging, and chimera removal. The FilterAndTrim function in DADA2 was used with standard parameters (maxN = 0, truncQ = 2, maxEE = 2) for sequence filtering. Open-reference clustering of features [50] and reference-based chimera filtering were performed using the SILVA (version 132) database at 99% similarity [51]. Rarefaction curve analysis was done to estimate the completeness of microbial community sampling. Based on the taxonomy generated, the feature-table was filtered to include only assigned reads of the kingdom Bacteria and to remove singleton features. Operational taxonomic units (OTUs) were presented as Venn diagrams. Comparison of Shannon and Simpson index between different groups was determined. Beta diversity was performed using Bray Curtis index, Jaccard distance matrix, and Unweighted Unifrac phylogenetic distance matrix [52–55]. Two-dimensional Principle Co-ordinated Analysis plots were generated to visualize bacterial community profiling. A PERMANOVA (α = 0.005) with 999 random permutations was performed to determine differences between groups.

### Bacterial load quantification

Total bacterial DNA, *Firmicutes* and *Bacteroidetes* load was quantified in gut and lung genomic DNA using Qiacuity digital PCR system (Qiagen, Hilden, Germany). Primer sequences are presented in Additional file 1: Table S1. Cycling conditions were set at 95 °C for 2 min (Cycle 1) followed by amplification cycles (40 cycles) at 95 °C for 15 s, 60 °C for 15 s, 72 °C for 15, followed by 1 cycle at 35 °C for 10 min. Non-template controls were run alongside the specimens. Thresholds were calculated based on the positive and negative RFU populations. Bacterial load is presented as copy number per ng of DNA.

### Short chain fatty acids preparation and analysis

Three major short chain fatty acids (SCFA) (acetate, propionate, and butyrate) were measured using serum samples. For GC/MS analysis, samples were prepared by adding 400 μL of 100 mM PFBBr acetone solution to 50 μL of serum in 1.5 mL micro centrifuge tubes. Crotonic acid solution (4 μL) diluted 100X in PBS was added to each of the samples. Similarly, standards were prepared by adding 400 μL of 100 mM PFBBr acetone solution to 24 μL of a mixture of C2, C3, and C4. Sample and standards (4 μL each) were transferred to 2 mL glass auto sampler vials and incubated at 70 °C for 1 h in a dry bath. Following incubation, the samples and standards were allowed to cool for 5 min, and 1 mL of hexane was added to the samples in the auto sampler vials. The samples and standards were transferred to new 1.5 mL micro centrifuge tubes and vortexed for 5 min and centrifuged at 300 RCF for 1 min. 200 μL of upper phase (hexane phase) was transferred to glass GC vials for further analysis.

The GC/MS data was obtained on a 5975C MSD equipped with a 7890A GC system. Separation was performed on a DB5-MS column (30 m × 0.25 mm × 0.25um) starting at 60 °C for 0.1 min, ramping 10 °C/min to 280 °C. Sample (2 μL) was injected in splitless mode. Negative chemical ionization spectra were obtained with methane as the chemical ionization gas. The source and quad temperatures were 150 °C. Full scan and selective ion monitoring (SIM) were acquired simultaneously. Full scan was performed in the range of 50–100 amu. Ions used for SIMS were 57, 73, 87 and 85. Integration was performed by taking the ratio of the SIM ion of interest peak area/SIM 85 peak area (internal standard). Diluted standards were utilized to produce a standard curve that was utilized for quantification of individual samples.

### RNA isolation/real-time PCR

RNA was isolated from tissue homogenates (lungs) to analyze gene expression at the mRNA level. Total RNA was isolated using the RNeasy Mini Kit (Qiagen) as per manufacturer’s instructions. Reverse transcription was performed using the High-Capacity cDNA Reverse Transcription Kit following manufacturer protocols (Thermo fisher Scientific Waltham, MA) and diluted to a working concentration of 10 ng/µL using nuclease free water. Real-time PCR was performed using the AriaMx Real-time PCR System (Agilent, Santa Clara, CA). Each PCR reaction mixture contained Syber Green® 12.5 µL, cDNA 5 µL, primers 3 µL and nuclease free water 2 µL with a total volume of 22.5 µL. The relative expression level was measured using comparative threshold method using Aria Real-Time PCR Software with 18S as the reference gene. Data were analyzed using the 2^−ΔΔCT^ method (Livak and Schmittgen. 2001). PCR primer sequences are provided in the Additional file 1: Table S1.

### Electron paramagnetic resonance (EPR) spectroscopy

Oxidizing potential of mouse serum, bronchoalveolar lavage fluid (BALF), and tissue (normalized to weight) were measured by EPR spectroscopy using 1-hydroxy-3-carboxymethyl-2,2,5,5-tetramethyl-pyrrolidine (CMH) spin probe (Enzo Life Science) as described previously [4, 56]. EPR spectra were recorded using a Bruker ELEXSYS E580 spectrometer (Bruker BioSciences, Billerica, MA, USA) operating at X-band with 100 kHz modulation frequency.

Briefly, serum/BALF samples and weight-normalized tissue homogenates were incubated with EPR spin probe CMH (200 μM) for 30 min at 37 °C. CMH (EPR inactive) is oxidized by reactive/oxidizing species in the serum/BALF/tissue homogenates to the EPR active 3-carboxymethyl-2,2,5,5-tetramethyl-pyrrolidinyloxy radical (CM•;). After incubation, samples were flash frozen in liquid nitrogen and stored at −80 °C until EPR analysis. At the time of EPR measurements, frozen samples were thawed to room temperature and immediately loaded (50 μL) into glass capillary tubes (Cat. No.: 2-000-050; Drummond Scientific Company Broomall, PA, USA). Capillary tubes were sealed on one end using Critoseal clay and placed inside 4 mm (O.D.) EPR quartz tubes. The quartz tube were placed inside the resonator/cavity and spectra were recorded at room temperature. The following EPR instrument settings were used, microwave frequency, 9.855 GHz; center field, 3495 G; sweep width, 100 G; microwave power, 23.77 mW; modulation amplitude, 1 G; modulation frequency, 100 kHz; receiver gain, 60 dB; conversion time, 14.65 ms, sweep time, 30 s; number of scans, 1. EPR data acquisition was performed using Bruker Xepr software. Signal intensity was generated using first peak (low field) height of the EPR spectrum. Data processing was performed using GraphPad Prism 9 software (GraphPad software, San Diego, CA).

### Hydrogen peroxide (H_2_O_2_) measurement

The hydrogen peroxide levels from whole lung homogenate were measured using Amplex™ Red Hydrogen Peroxide/Peroxidase Assay Kit (Thermo Fisher Scientific Waltham, MA) following manufacturer’s instructions. Briefly, tissues were pulverized at −80 °C followed by lysis using RIPA buffer supplemented with protease inhibitor (Sigma-Aldrich, St. Louis, MO). Samples were then incubated with Horseradish peroxidase (HRP) and Amplex Red dye. The red-fluorescent oxidation product, resorufin, was produced by the reaction of the Amplex Red fluorescent dye and H_2_O_2_ from the samples. The SpectraMax® iD5 (Molecular Devices, CA) plate reader was used to measure the absorbance after 45 min incubation, at 560 nm wavelength. The results are normalized to total protein concentration measured by BCA (bicinchoninic acid) assay kit as described previously [3, 57].

### Statistical analysis

The statistical analysis was performed with GraphPad Prism 9 (GraphPad Software, San Diego, USA). Data are presented as mean ± standard error of mean. For normally distributed data, we performed two-way ANOVA followed by Tukey’s post hoc test for statistical analysis (comparison between one day and four days exposures). Shapiro–Wilk test was performed for analyzing the data distribution. Non-normally distributed data were evaluated using Two-way ANOVA followed by Kruskal–Wallis post-test. We performed One-way ANOVA for only those data sets where only one time point was performed (comparison only required to test for differences between treatments). Statistical significance was considered at *p* ≤ 0.05.

Beta diversity metrics (Jaccard distance, Bray–Curtis distance, Unweighted UniFrac distance) and generated principal coordinates analysis PCoA plot were calculated using Emperor [58] for each of the beta diversity metrics. Group significance between alpha and beta diversity indexes was calculated with QIIME2 plugins using the Kruskal–Wallis test and permutational multivariate analysis of variance (PERMANOVA), respectively. The relative abundance with respect to taxonomy phylum were visualized using GraphPad [59] (GraphPad Prism version 9).

## Results

### Exposure characteristics and mice weight

Stable aerosol generation was confirmed by the real-time monitoring of CB particles and O_3_ levels over the exposure period. Gravimetric measurements for the exposure period indicated the achievement of the desired concentrations [CB one day (10.33 ± 0.80 mg/m^3^), CB four days (10.62 ± 0.99 mg/m^3^), O_3_ one day (2 ± 0.06 ppm), O_3_ four days (1.99 ± 0.06 ppm), CB + O_3_ one day (CB 10.51 ± 0.08 mg/m^3^, O_3_ 1.77 ± 0.5 ppm), CB + O_3_ four days (CB 10.97 ± 0.54 mg/m^3^, O_3_ 1.97 ± 0.26 ppm)]. Aerosol size distributions indicated that the majority of particles were in the nano-size range with count median diameters of 82.9 nm and 84.5 nm and mass median diameters of 0.90 nm and 0.97 nm for CB and CB + O_3_, respectively. ELPI + measurements, based on charge has median diameters of 64.5 nm and 74.4 nm (Additional file 1: Table S2).

We have collected weight data and that indicated a slight decrease (~ 5%) in weight after first exposure but that did not reach statistical significance (Additional file 1: Figure S1). Moreover, repeated exposures did not add any further loss in weight.

### Lung immune cell profiling

Whole lung flow cytometry was performed to analyze the inflammatory cell influx in the exposed animal lungs. Multiple exposures to CB + O_3_ induced a significant (*p* = 0.03) increase in the number of neutrophils (EPCAM^−^, CD45^+^, Ly6G^+^) (Fig. 1B). Eosinophil (EPCAM^−^, CD45^+^, Ly6G^−^, CD11c^−^, SiglecF^+^) numbers also increased after multiple exposures to CB + O_3_. Increased numbers of lung eosinophils were also observed after multiple exposures to CB or O_3_. Single CB exposure, and multiple exposures to either CB, O_3_, or CB + O_3_, increased B lymphocyte numbers. T cell (Epcam^−^, CD45^+^, CD3e^+^) numbers decreased significantly after single or multiple exposures to O_3_ and CB + O_3_. Raw values and animal numbers used to generate the heat map are presented in Additional file 1: Table S3.

### Features of lung microbiome after inhalation exposure

A total of 1,412,598 features were obtained from the lung samples. Sequences were then de-noised, demultiplexed, and chimera filtered (accounting for 25.62% of the raw sequences). Sequences were classified into OTUs (at 97% similarity). OTUs were categorized into 9 phyla, 13 classes, 33 orders, 58 families, and 91 genera, by comparing to SILVA 132 database.

#### Community profile of the lung microbiome

**Fig. 2:**
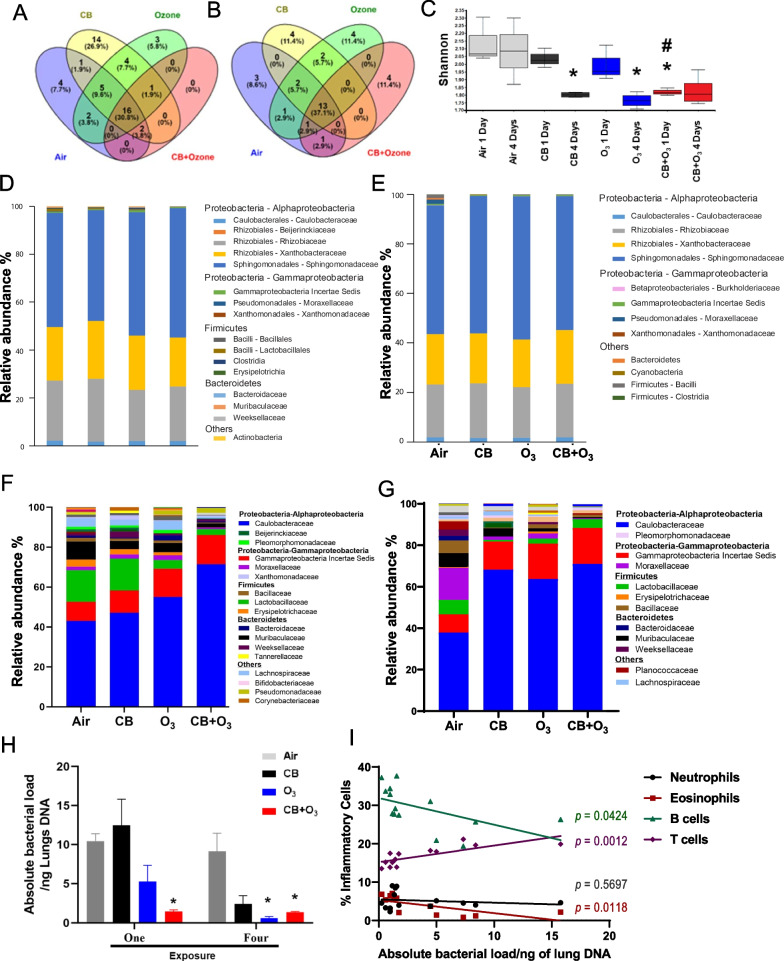
Alterations in the lung microbiome. **A** Unique alterations in OTUs after single exposure **B** Family level analysis of shared and unique OTUs after multiple exposures **C** Shannon alpha diversity index. The boxplots show median, quartile, smallest and largest observations. **D** Bacterial taxonomic profiles at the family level in the lung microbiota after single day CB, O_3_ or CB + O_3_ exposure. **E** Bacterial taxonomic profiles at the family level in the lung microbiota after multiple days CB, O_3_ or CB + O_3_ exposure. **F** Bacterial taxonomic profiles (family level) after removing top three families (single exposure). **G** Bacterial taxonomic profiles (family level) after removing top three families (multiple exposures). **H** Absolute quantification of bacterial load per ng of lung DNA was analyzed using Qiacuity digital PCR. **I** Correlation between absolute bacterial load in the lungs and inflammatory cells (neutrophils, eosinophils, B and T cells) after multiple exposures. *p ≤ 0.05. *denotes significantly difference compared to air, ^**#**^denotes significantly difference compared between ozone alone and co-exposure. Data are presented as mean ± standard error of mean (SEM) and analyzed by Two-way ANOVA followed by Tukey’s post hoc test. The Kruskal–Wallis test was performed to determine the statistical significance of Shannon diversity index n = 3–5

We evaluated whether exposure alters the microbial community in the lungs. After single exposure, the majority of unique OTUs (family level) were shared by the different exposure conditions (Fig. 2A) and only a few were uniquely present in individual exposures while none was unique to co-exposure. After multiple co-exposures, the number of bacterial families uniquely present in the lung microbiome increased while those uniquely present in the air, CB, and O_3_ decreased (Fig. 2B). An increase here refers a comparison between single and multiple exposure and the number of families points towards a magnitude of the change. The shared and unique OTUs (family level) in response to single exposure and multiple exposures are shown in Table 1 indicating the shifting of the bacterial community in response to environmental exposure. *Acetobacteraceae* family was observed in the control sample uniquely whereas, *Halomonadaceae*, *Prevotellaceae* and *Clostridiaceae 1* were observed uniquely after multiple CB + O_3_ exposure. We also analyzed alpha diversity to calculate community diversity and richness based on the OTUs. Single CB or O_3_ exposure did not reduce the Shannon index significantly, while co-exposure showed a significant reduction in index value compared to control and other individual exposures (Fig. 2C). Multiple exposures to CB and O_3_ alone reduced alpha diversity significantly (*p* = 0.02 and *p* = 0.03, respectively). Multiple exposures of CB + O_3_ also reduced the Shannon diversity index (*p* = 0.07).

**Table 1:** Shared and unique families after air, CB, O_3_ or CB + O_3_ exposure in lungs

Shared		Unique			
Day 1	Day 4	Day 1			
		Air	CB	O_3_	CB + O_3_
*Sphingomonadaceae*	*Sphingomonadaceae*	*Acetobacteraceae*	*Streptococcaceae*	*Thioalkalispiraceae*	None
*Rhizobiaceae*	*Rhizobiaceae*	*Enterococcaceae*	*Helicobacteraceae*	*Family XI*	
*Xanthobacteraceae*	*Xanthobacteraceae*	*Clostridiaceae 1*	*Paenibacillaceae*	*Atopobiaceae*	
*Caulobacteraceae*	*Caulobacteraceae*	*Chloroplast*	*Nocardiaceae*		
*Gammapro Incertae Sedis_Un*	*Gammapro Incertae Sedis_Un*		*Microscillaceae*		
*Lactobacillaceae*	*Lactobacillaceae*		*Dongiaceae*		
*Moraxellaceae*	*Moraxellaceae*		*Nostocaceae*		
*Muribaculaceae*	*Muribaculaceae*		*Halomonadaceae*		
*Bacillaceae*	*Bacillaceae*		*Phaseolus acutifolius*		
*Lachnospiraceae*	*Lachnospiraceae*		*Family X*		
*Weeksellaceae*	*Beijerinckiaceae*		*Micrococcaceae*		
*Xanthomonadaceae*	*Pleomorphomonadacea*		*Alteromonadaceae*		
*Ruminococcaceae*	*Rhizobiales Incertae Sedis*		*Deinococcaceae*		
*Pseudomonadaceae*			*Deferribacteraceae*		
*Erysipelotrichaceae*					
*Bacteroidaceae*					

Day 4			
Air	CB	O_3_	CB + O_3_
*Bacteroidaceae*	*Chloroplast*	*Erysipelotrichaceae*	*Prevotellaceae*
*Ruminococcaceae*	*Mitochondria*	*Tannerellaceae*	*Clostridiaceae 1*
*Stappiaceae*	*Devosiaceae*	*Neisseriaceae*	*Halomonadaceae*
	*Armatimonadales*	*URHD0088*	*Rikenellaceae*

#### Composition of the lung microbiome

At the phylum level, *Proteobacteria* was the prominent phylum (more than 90%) in the lungs followed by *Firmicutes*, *Bacteroidetes*, *Actinobacteria*, and *Cyanobacteria*. The level of *Proteobacteria* decreased after single or multiple CB + O_3_ co-exposure (Fig. 2D, E). We also analyzed the relative abundance of the bacterial composition at the family level and found Sphingomonadaceae*, Rhizobiaceae*, *Xanthobacteraceae*, *Lactobacillaceae*, and *Murribaculaceae* as the most abundant families (Fig. 2D, E). The taxonomic composition of bacterial families in the lungs was better visualized by removing the top three families in Fig. 2F, G. We found increases in *Caulobacteraceae* and *Gammaproteobacteria incertae sedis* and a reduction in *Pleomorphomonadaceae* and *Moraxellaceae*. *Lactobacillus* showed a trend toward reduction after inhalation exposure to CB and O_3_ (Additional file 1: Figure S3A).

Total bacterial DNA analyzed by Qiacuity One digital PCR in the lung also showed a reduction compared to the control (Fig. 2H); single co-exposure reduced total bacterial load in the lungs compared to any other groups. Moreover, multiple exposures to CB, O_3_, and CB + O_3_ also reduced the total bacterial population compared to the control group. In our results, Bray–Curtis and Unweighted Unifrac did not show significant differences among groups in the lungs (Additional file 1: Figure S3B, C). We have performed correlation analyses between inflammatory cell numbers and absolute bacterial/microbial load. We found significant correlation between CB + O_3_ co-exposure induced increase in eosinophils in the lungs and total bacterial load (Fig. 2I). Reduction of absolute load was also significantly correlated with increased B cells (Fig. 2I). In addition, an inverse correlation exists between T cell numbers in the lungs and total bacterial load (Fig. 2I).

### Features of gut microbiome after inhalation exposure

#### Diversity profile of colon microbiome

**Fig. 3:**
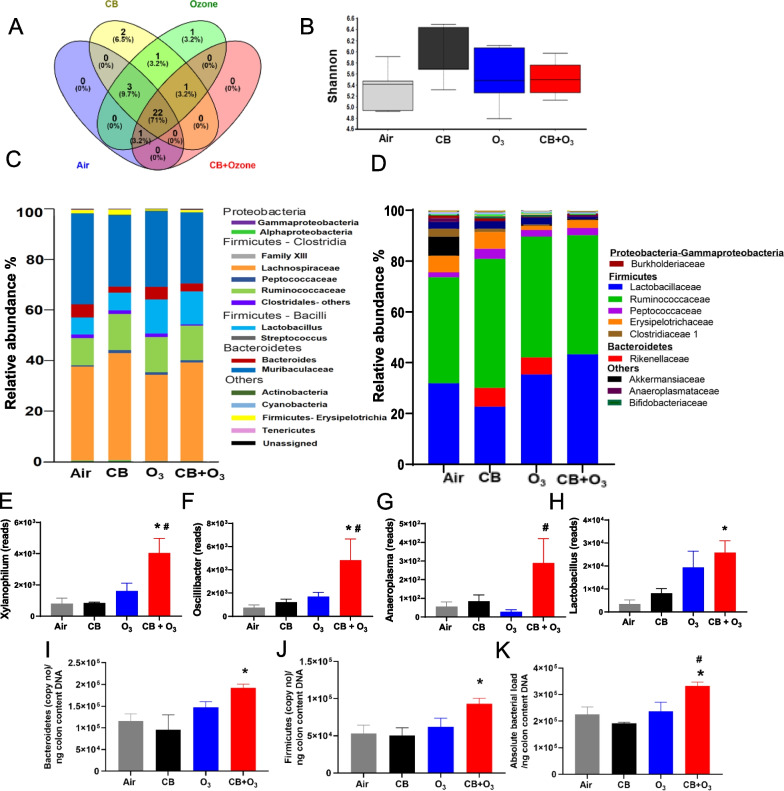
Alterations of alpha diversity indices and relative abundance of gut microbial communities after multiple exposures. **A** Family level analysis of shared and unique microbiota at multiple exposures showing altered unique OTUs compared to air. **B** Shannon diversity index. The boxplots show median, quartile, smallest and largest observations. **C** Bacterial taxonomic profiles at the family level in the colon microbiota after multiple days CB, O_3_ or CB + O_3_ exposure. **D** Bacterial taxonomic profiles (top three families removed) after multiple exposures. **E–G** Significant increase in *Xylanphilum*, *Oscillibacter* and *Anaeroplasma* in the colon contents. **H** *Lactobacillus* significantly increased after multiple CB + O_3_ co-exposure. **I** *Bacteroidetes* level in the gut DNA increased significantly compared to control. **J** *Firmicutes* level altered in the gut. **K** Absolute bacterial load per ng of colon content DNA was analyzed using Qiacuity digital PCR. *p ≤ 0.05. *denotes significantly difference compared to air. ^#^denotes significantly difference between ozone and co-exposure. The Kruskal–Wallis test was performed to determine the statistical significance of diversity index. n = 5–6

A total of 2,292,698 features were obtained in the gut samples. Sequences were then denoised, demultiplexed, and chimera filtered. Sequences were categorized into 7 phyla, 11 classes, 16 orders, 32 families, and 84 genera by comparing them to the SILVA database. We evaluated the shared and unique OTUs in the gut samples at the family-level which showed alterations of unique OTUs among treatment groups (Fig. 3A). The shared and unique family level OTUs in response to exposure are listed in Table 2. To measure bacterial alpha diversity, we measured the Shannon index. Count-based Shannon index did not show significant alteration between the exposure groups (Fig. 3B).

**Table 2:** Shared and unique families after multiple exposures of air, CB, O_3_ or CB + O_3_ in gut contents

Shared OTUs	Unique OTUs			
	Air	CB	Ozone	CB + Ozone
*Lactobacillaceae*	None	*Rhodospirillales_Uncultutred*	Enterococcaceae	None
*Muribaculaceae*		*Caulobacteraceae*		
*Bacteroidaceae*				
*Lachnospiraceae*				
*Ruminococcaceae*				
*Peptococcaceae*				
*Erysipelotrichaceae*				
*Clostridiaceae 1*				
*Clostridiales vadinBB60 group*				
*Anaeroplasmataceae*				
*Burkholderiaceae*				
*Mitochondria*				
*Sphingomonadaceae*				
*Mollicutes RF39*				
*Eggerthellaceae*				
*Family XIII*				
*Defluviitaleaceae*				
*Rhizobiaceae*				
*Mollicutes RF39*				
*Xanthobacteraceae*				
*Chloroplast*				
*Clostridiales*				
*Christensenellaceae*				

#### Effect of exposure on colon microbiome composition

The most abundant bacterial phyla were *Proteobacteria*, *Firmicutes*, and *Bacteroidetes* in the gut microbiome. Family-level analysis of microbial communities revealed an increased relative abundance of *Lactobacillaceae* in multiple O_3_ and CB + O_3_ exposure*. Ruminococcaceae* was also increased in all three exposure groups (CB, O_3_ and CB + O_3_) in the gut microbiome (Fig. 3C). In addition, decreased levels of *Clostridiaceae I* were observed. To better visualize taxonomic profiles, relative abundance was presented by removing the top three families in Fig. 3D.

At the genus level, *Xylanophilum*, *Oscillibacter*, and *Anaeroplasma* were increased significantly in CB + O_3_ compared to control and O_3_ alone (Fig. 3E–G). *Lactobacillus* was significantly increased in CB + O_3_ exposed mice and a trend was found in O_3_ only exposure (*p* = 0.13) (Fig. 3H). Relative abundance of *Bifidobacterium* were also increased after CB alone and CB + O_3_ exposure. We found no significant difference in beta diversity indices (Bray-Curits, Jaccard dissimilarity, and Unweighted Unifrac) due to CB + O_3_ co-exposure in the colon content microbiome (Additional file 1: Figure S4A-C).

#### Bacterial load in the gut

We observed a significant increase in *Bacteroidetes and Firmicutes* in CB + O_3_-exposed groups after multiple exposures (Fig. 3I, J). The *Firmicutes* to *Bacteroidetes* ratio did not alter due to CB and O_3_ co-exposure (Additional file 1: Figure S4D). Multiple exposures to CB + O_3_ resulted in a significant increase in the absolute bacterial load in the gut (Fig. 3K).

### Oxidant stress response

**Fig. 4:**
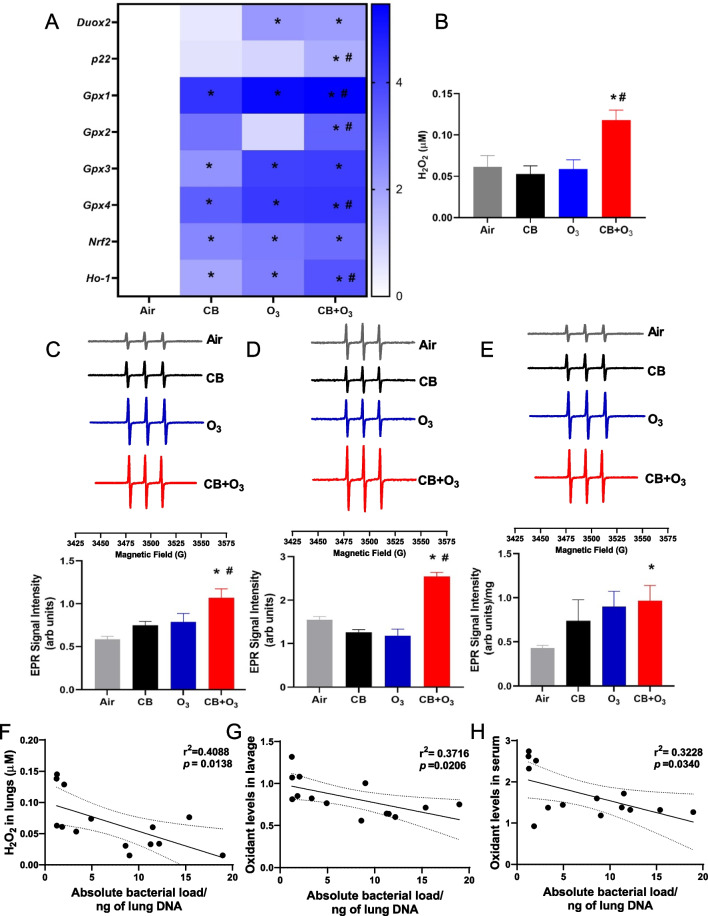
Alterations in oxidative stress response. **A** Alterations in Duox*2*, *p22*, *Gpx1*, *Gpx2*, *Gpx3*, *Gpx4*, *Nrf2*, and *Ho-1* gene expression in lungs after single air, CB, O_3_ and CB + O_3_ exposure. **B** Significant increase in H_2_O_2_ levels in the lungs after single co-exposure. **C** Representative room temperature X-band EPR spectra of CM^•^ radical in bronchoalveolar lavage fluid after single exposure, and plot of EPR signal intensity **D** Representative room temperature EPR spectra of CM^•^ radical in serum after single exposure, and plot of signal intensity. **E** Representative room temperature EPR spectra of CM^•^ radical in gut samples after multiple exposures and plot of EPR signal intensity. **F** Correlation of absolute bacterial load in the lungs to hydrogen peroxide levels after single exposure. **G-H** Correlation of absolute bacterial load in the lung and redox levels in the lavage and serum, respectively after single inhalation exposure. Gene expression data are presented as log fold changes compared to 18 s (internal control). Data are presented as mean ± standard error of mean (SEM) and analyzed by One-way Analysis of Variance (ANOVA) followed by Tukey’s post hoc test. n = 5–6. *p ≤ 0.05. *denotes significantly difference from air, and ^#^ denotes significantly different from O_3_ at the same time point

Oxidant stress is an early mediator of exposure-induced changes, with changes in oxidant parameters observed after a single exposure. In the lungs, *Duox2* expression was significantly increased by single O_3_ and CB + O_3_ exposure (Fig. 4A). *P22* expression in the lungs was increased only after co-exposure to CB + O_3_ exposure. *Gpx1*, *Gpx3*, and *Gpx4* expressions were significantly increased after co-exposure and individual CB and O_3_ exposure. However, co-exposure induced significantly greater expression of *Gpx1*, *Gpx2*, and *Gpx4* compared to O_3_ exposure alone*. Gpx2* was only induced by co-exposure and *Gpx3* was induced to a similar extent by all the exposures. *Nrf2* and *Ho-1* expression was significantly induced in all three exposures. However, CB + O_3_ exposure induced significantly greater *Ho-1* expression compared to individual exposures. Heat map statistics for mRNA expression was presented in Additional file 1: Table S4. Lung tissue H_2_O_2_ quantification by the Amplex Red assay also confirmed significantly greater oxidant production after CB + O_3_ exposure (Fig. 4B). Greater pulmonary and systemic oxidant production after co-exposure was further confirmed by the EPR analyses on the lavage, serum, and gut tissues (Fig. 4C–E). We observed significant inverse correlation between total bacterial load in the lungs and H_2_O_2_ levels (Fig. 4F). Similarly, an inverse correlation was observed between lung total bacterial load and lavage and serum oxidant levels (Fig. 4G, H). Correlation between absolute bacterial load in the colon contents and colon redox potential is presented in Additional file 1: Figure S5.

### Systemic mediators of microbiome alterations

#### SCFA analyses

**Fig. 5:**
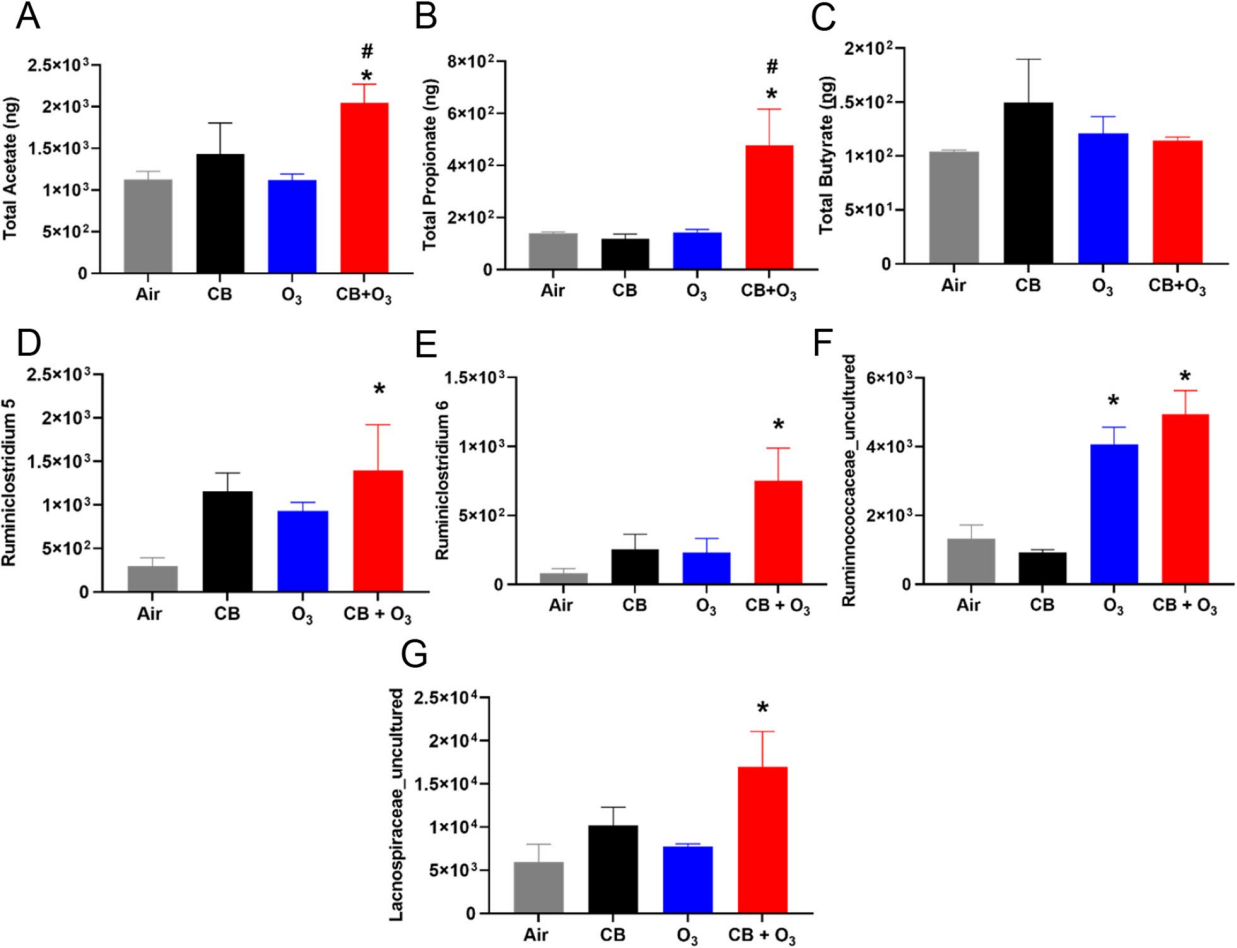
Alterations in short chain fatty acid content and respective microbiome. **A** Alterations in serum acetate after multiple exposures. **B–C** Alteration in propionate and butyrate content in serum by GC–MS after multiple exposures. **D–G** Significant increase in *Ruminiclostridium 5*, *Ruminiclostridium 6*, *Ruminococcaceae_uncultured* and *Lacnospiraceae_uncultured* in the colon content after multiple CB + O_3_ exposure. Data are presented as mean ± standard error of mean (SEM) and analyzed by One-way Analysis of Variance (ANOVA) followed by Tukey’s post hoc test. n = 5–6. *p ≤ 0.05. *denotes significantly difference from air, ^#^ denotes significantly different from O_3_ at the same time point

Serum SCFAs (acetate, butyrate, and propionate) analyses by GC–MS indicated a significant increase in the acetate levels after CB + O_3_ exposure (multiple exposures) (Fig. 5A). Propionate levels were only increased after multiple CB + O_3_ exposures (Fig. 5B). Butyric acid levels were not altered by the exposures (Fig. 5C). Next, we analyzed SCFA-producing bacterial abundance in the gut and observed a significant increase in *Ruminiclostridium 5, Ruminiclostridium 6, Ruminnococcaceae-uncultured,* and *Lacnospiraceae_uncultured* (Fig. 5D–G), which are prominent producers of acetate and propionate.

#### Altered expression of gut-derived metabolic mediators

**Fig. 6:**
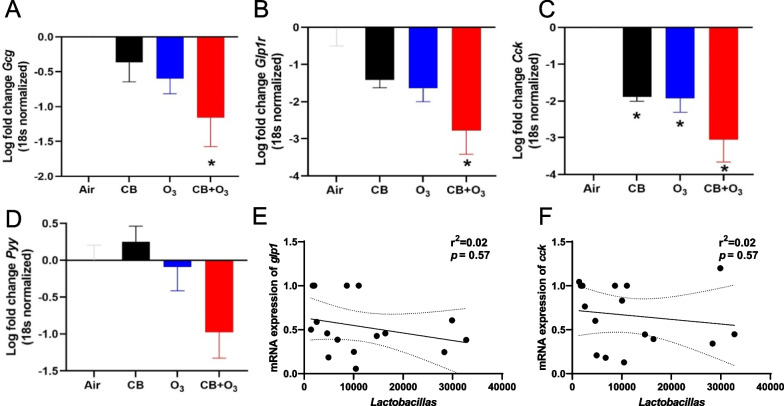
Alterations in gut microbiome produced secondary mediators. **A** Alteration in the *Gcg* mRNA expression after multiple exposures in the colon as log fold change, **B–D** Alteration in the *Glp1r*, *Cck* and *Pyy* mRNA expression after multiple exposures in the colon as log fold change. **E, F** Correlation of *Glp1r* and *Cck* mRNA expression and *Lactobacillas* abundance after multiple exposures in the colon. Data are presented as mean ± standard error of mean (SEM) and analyzed by One-way Analysis of Variance (ANOVA) followed by Tukey’s post hoc test. n = 5–6. *p ≤ 0.05. *denotes significantly difference from air

Real-time PCR-based gut mRNA expression of *Gcg* (proglucagon), *Glp1r* (glucagon-like peptide 1 receptor), and *Cck* (cholecystokinin) was significantly reduced after multiple exposures to CB + O_3_ (Fig. 6A–C). *Pyy* (peptideYY) expression also demonstrated a trend toward down-regulation (Fig. 6D). Moreover, a significant inverse correlation exists between the read counts of beneficial bacteria (*Lactobacillus)* and *Glp1r* and *Cck* gene expression in the gut tissue (Fig. 6E, F).

## Discussion

**Fig. 7:**
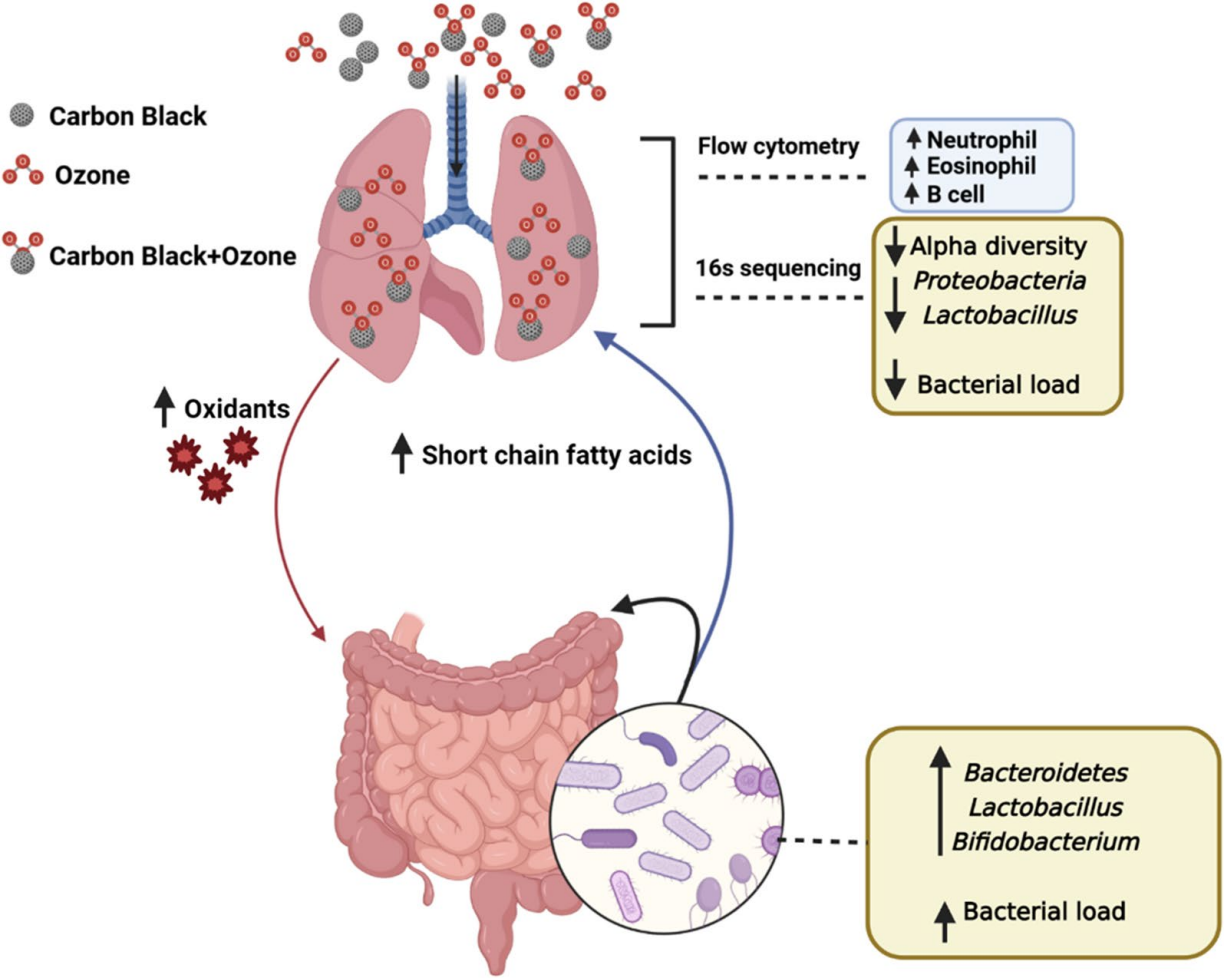
Overview figure representing co-exposure induced pulmonary and intestinal alteration. Neutrophils, eosinophils and B cells were significantly increased in the lungs after multiple CB + O_3_ co-exposure. Microbial alpha diversity and total bacterial load were reduced in the lungs. CB + O_3_ exposure also significantly induced serum short chain fatty acids (SCFA) and oxidant level (BALF, serum and gut tissue). Gut total bacterial load, *Lactobacillus* and *Bididobacterium* significantly increased after multiple CB + O_3_ exposure

This study validated that unique lung and gut microbiome alterations occur following inhalation co-exposure to CB and O_3_. The impact of environmental exposure was evident from the reduction in microbial diversity in the lungs. Conversely, an increased abundance of certain beneficial bacteria without significant alterations in the richness and diversity of the overall microbiome in the gut indicated a complex, but potentially homeostatic, shift to counter the adverse impacts of inhalation exposure. Gut microbiome-derived SCFA alterations further support this notion. An increased oxidant stress in the lungs, serum, and gut tissue after co-exposure indicated a potential systemic spillover from inhalation exposures. Systemic impacts of inhalation exposure were further supported by the changes in gene expression of gut-derived metabolic mediators (*Gcg* and *Pyy*). An overview of these changes is presented in Fig. 7.

We demonstrated a significant increase in neutrophils, eosinophils, and B cells after inhalation exposure to CB + O_3_. We previously reported increased total cells, neutrophils, and macrophages in the BALF after CB + O_3_ co-exposure [3, 4]. In the present study, we extended these findings using lung tissue cell typing by flow cytometry and demonstrated a significantly greater increase in neutrophils, eosinophils, and B lymphocytes after repeated CB + O_3_ co-exposure compared to individual exposures. Moreover, we observed a significant increase in eosinophils and B cells in the lung tissue after multiple exposures to CB, O_3,_ and CB + O_3_. Exacerbation of eosinophilic airway inflammation was reported after O_3_ exposure in mice [60]. An increase in lung eosinophils after single and repetitive O_3_ inhalation exposures has also been previously reported [61–63]. Increased numbers of eosinophils were also previously reported after diesel exhaust exposure in humans [64]. We observed a significant decrease in T cell numbers in lung tissue after inhalation exposures to O_3_ and CB + O_3_. T-lymphocytes are an important part of the adaptive immune response.

16 s rRNA sequencing analysis of the lung microbiome demonstrated significant alterations in microbiome richness and abundance. Shannon diversity index decreased significantly after single CB + O_3_ exposure, indicating a significantly greater ability of acute exposure to CB + O_3_ to reduce microbial richness compared to individual CB or O_3_ exposures. A previous study reported a reduction of alpha diversity indices (Shannon and Fisher) after PM_2.5_ exposure in mice [65]. Moreover, human studies have also shown reduced Shannon and ACE index after exposure to O_3_ [66]. The observation of unique bacterial families point towards divergent proposed functions in the lung pathophysiology. Potentially beneficial *Acetobacteraceae* family members were only observed in the control group. *Acetobacteraceae* is known to have beneficial impacts in terms of attenuating airway allergic inflammation in mice [67]. On the other hand, after multiple co-exposures, several bacterial families that are associated with different pathologies (*Prevotellaceae, Halomonadaceae, Clostridiaceae*) were observed in the lungs [65, 68, 69]. In terms of relative abundance, we observed an increase in *Caulobacteraceae* and *Gammaproteobacteria incertae sedis* in response to single and multiple CB + O_3_ co-exposures. An increase in *Caulobacteraceae* in lungs in response to ambient PM exposure in rat has been reported [70]. Human studies regarding COPD and lung adenocarcinoma have also been reported to have increased *Caulobacteraceae* [71, 72]. We also found a significant reduction in *Lactobacillaceae* in the lungs.

A bidirectional relationship is proposed to exist between inflammation and microbiome. Inflammatory cells and milieu have been shown to change the composition of microbiome [73]. Alternately, various microbial products act on pattern recognition receptors and have potential to activate various inflammatory cells/pathways [74]. SCFA such as acetate and butyrate exert anti-inflammatory impacts by modulating of g-protein coupled receptor signaling, NF-kB or Interferon-gamma levels [75–78]. Relationship between inflammation and microbiome has been extensively reviewed elsewhere [75, 79]. We further performed correlation analyses between inflammatory cell numbers and absolute bacterial/microbial load. We found significant linear negative correlation between CB + O_3_ co-exposure and eosinophils and B cells while a positive correalton was observed for T cell. Clinical and in vivo studies also report a correlation between bacterial load and exacerbation of inflammatory cells and disease phenotype [80, 81].

In analyzing beta diversity, which provides differences in microbial communities between samples, we found that the dominant communities are similar among treatment groups. This observation is supported by previous studies demonstrating no change after PM exposures and in pneumococcal infections [82, 83].

Total gut bacterial load was significantly increased after multiple CB + O_3_ exposures. However, there was no change in the Shannon richness index in the gut microbiome after multiple exposures to CB, O_3,_ or CB + O_3_. Conflicting reports about gut microbial diversity exist after inhalation exposure, demonstrating either an increased or decreased gut microbiome abundance after PM exposure [42, 67, 84, 85]. Several significant experimental design and technical differences (mode and duration of exposure, mouse strains, and techniques to assess the microbiome) are potential reasons for these conflicting findings. In our gut microbiome analysis, *Firmicutes*, *Bacteroidetes and Proteobacteria* were the most abundant bacteria while *Verrucomicrobia*, and *Actinobacteria* were less abundant. On multiple occasions, the changes in bacterial populations in the gut were indicative of unique and opposite biological outcomes compared to the lungs. For example, a significant increase in *Oscillibacter* was observed in the co-exposure group compared to controls and O_3_ alone. *Oscillibacter* abundance is negatively correlated with transepithelial resistance [86]. On the other hand, an increased relative abundance of *Anaeroplasma* was observed which is associated with improved barrier function in the intestine [87]. We also observed increases in potentially beneficial gut bacteria after inhalation exposure. The presence of certain bacteria known to function in maintaining host immunomodulatory balance such as *Lactobacillus, Enterococcus faecalis, Streptococcus,* and *Bifidobacterium* indicates a potential adaptive mechanism of the gut microbiome [88–90]. Overall, this indicates that inhalation co-exposure imbalances gut microbial composition toward a potential adaptive/homeostatic change.

To assess the systemic mediators of lung and gut microbiome dysbiosis, we evaluated secondary metabolites (*e.g.,* SCFA) produced by the gut microbiome and peptides produced by the intestinal cells (*e.g.,* GLP-1, CCK). Acetate, propionate, and butyrate are the three most important SCFAs that regulate tissue immune homeostasis [91]. We observed a significant increase in acetate levels after multiple co-exposures. Propionate levels were also increased after multiple CB + O_3_ co-exposures. Increased acetate and propionate contents in the serum are in line with previously reported increases after rodent PM exposure [92]. A significant increase in prominent acetate and propionate producers in the gut (*Ruminiclostridium5*, *Ruminiclostridium6*, *Ruminococcaceae_uncultured*, and *Lachnospiraceae_uncultured*) further indicates a potential source for these SCFAs. We did not observe any change in the serum butyrate contents.

*Gcg* (Glucagon) regulates glucose metabolism and homeostasis and is reported to be linked with gut microbiota [93]. We observed a significant reduction in *Gcg* mRNA expression after multiple CB + O_3_ co-exposures. *Glp1r* is a G-protein coupled receptor for glucagon-like peptide 1 (GLP-1). GLP-1 exerts metabolic effects including GI motility, the release of insulin, and suppression of appetite. Significant reduction in *Glp1r* and *Cck* expression was found in the gut samples after multiple CB + O_3_ exposures. These finding are in line with previous studies showing reduced gut *Gcg* expression and subsequent reduced GLP-1 contents [93, 94]. Cumulatively, these findings point towards an adverse impact of environmental exposures especially co-exposure to CB and O_3_ in the metabolic disorders such as type 2 diabetes and obesity. Further in-depth mechanistic evaluations will be needed to clarify implicated pathways.

We and others have previously reported redox imbalance as an early event and a critical mediator of O_3_ and CB + O_3_ induced pulmonary inflammation and injury [3, 4]. Lung and systemic redox imbalance were evident from changes in BALF, serum, and gut tissue oxidant levels (EPR and Amplex Red based analyses). CB + O_3_ co-exposure demonstrated increased potency compared to individual exposures to induce redox imbalance. This is in line with previously published findings of increased oxidant potential of CB + O_3_ compared to individual CB and O_3_ exposures [3, 4, 95]. Moreover, we observed significant inverse correlation between total bacterial load in the lungs and H_2_O_2_ levels. Similarly, an inverse correlation was observed between lung total bacterial load and lavage and serum oxidant levels. Previous studies have reported a link between oxidative stress, gut microbiome and various systemic diseases [96, 97]. This relationship is double edged sword. Physiological oxidant generation occur because of interaction of microbiome with intestinal epithelium [98]. These physiological levels of oxidants act as second messenger for various inflammatory signaling cascades [98]. However, these oxidants change the composition and functionality of the microbiome as anaerobes flourish in the presence of electron acceptors [99]. Moreover, this may result in the change in permeability of the intestine and altered bioavailability of microbial products and xenobiotic molecules [99]. Indeed, activation of formyl peptide receptor by bacterial products is known to induce inflammation and results in barrier dysfunction [97, 100]. Alternately, healthy microbiome and probiotic species have an immense anti-oxidative and anti-inflammatory role [97]. Finally, gut microbiota improves antioxidant function through absorbable vitamin, SCFA, polyphenols [97].

This work represents a solid step towards identifying the directionality of inflammation and microbiome alterations, potential intermediates or pathways involved or changed in case of environmental exposures. In this work we have defined changes in lung and gut microbiome, levels of oxidants (lung, systemic circulation, and gut), systemic levels of microbiome derived mediators (SCFAs) and gene expression levels of gut derived metabolic mediators. Further studies will mechanistically establish the role of these mediators in deriving inflammation/microbiome change. There are components of this proposed approach that are established such as the link between particle exposures and inflammation [101], oxidant exposures and lung inflammation [102], microbiome changes and SCFA level changes [103]. Our overview figures (Fig. 7) links various aspects of the current study showing potential connection of lung-gut axis.

The lung-gut axis implies that a potential connection of events in the respiratory tract and gastrointestinal tract exists and perturbation of one system may affect the other. Pulmonary exposure-induced systemic manifestations such as inflammation/immune alterations and oxidant stress can potentially impact the composition of the gut microbiome [104, 105]. Conversely, gut microbiota-induced immune alterations and systemically released mediators (i.e. SCFAs) can have an impact on lung inflammation or outcomes after inhalation exposure [30, 52]. Thus, the direction of interaction between gut and lung microbiota remains unclear [106]. An increased inflammatory response after inhalation exposure in the lungs after antibiotic-mediated gut microbiome depletion was previously reported [107]. We observed a significant increase in the abundance of *Lactobacillus*, *Ruminococcaceae_uncultured* and *Bifidobacterium* in the colon contents after inhalation exposures. Increase in *Lactobacillus* and *Bifidobacterium* was demonstrated to be associated with increases in serum SCFA concentration [108]. Previous literature reported that oral administration of *Lactobacillus* and *Bifidobacterium* ameliorated PM_2.5_-induced inflammatory cell migration and activation of pro-inflammatory cytokines [84, 109].

Our short duration exposure result in a deposition level that has relevance for both environmental and occupational exposures. Short exposure duration was preferred to avoid extended food and water restriction that could have caused nonspecific metabolic and microbiome alterations. This resulted in exposing mice to relatively higher number concentrations of particles. Still the deposited levels are well below the overload conditions [110]. We previously experimentally determined that the exposure regime used in this study (3 h) lead to a pulmonary deposited dose of 2.2 μg after a single exposure [3]. Moreover, at 24 h post exposure the remaining lung burden was 1.5 μg [111]. The Occupational Safety and Health Administration (OSHA) airborne permissible exposure limit for ultrafine CB is 3.5 mg/m^3^ over an 8-h work shift. Multiple Path Particle Dosimetry Model (MPPD v3.04) predicted 12.4% human pulmonary deposition fraction for CB exposure [112, 113]. This results in a deposited dose of 4.17 mg.

Following calculations were previously reported by us [4].

Factored for human dose using OSHA PEL of 3.5 mg/m.^3^

$$Aerosol\;concentration \times min\;volume \times exposure\;duration\;deposition\;efficiency = deposited\;human\;dose.$$

3.5 mg/m^3^ × (20L/min) (10^−3^ m^3^/L) × (8 h/day) × 60 min/h × 0.124 = 4.17 mg deposited/8 h in a worker.

Human equivalent to mouse measured deposited dose by surface area (SA):

$$\left( {SA_{human} \times \, Lung\;Burden_{mouse} } \right)/SA_{mouse} = \, Lung\;Burden_{human}$$

(102 m^2^ × 0.0022 mg)/0.05 m^2^ = 4.5 mg.

Thus, this deposited dose is not significantly different from a work deposited dose after single work shift at 3.5 mg/m^3^ for 8 h. This deposited dose is equivalent an anticipated deposited dose from a 35-day exposure of 35 μg/m^3^ national ambient air quality standard (NAAQS) for PM2.5 for 24 h. These levels are relevant for environmental exposures as global PM2.5 average levels routinely exceed 35 μg/m^3^ for approximately 90% of the urban population [114]. Rodent O_3_ require considering the factors such as anatomical differences and sedentary nature while exposures that result in higher (4–5 times) concentration exposures to achieve similar biological outcomes as observed in exercising humans after controlled ozone exposures [62, 115–119]. Accounting for these differences, we exposed animals to 2 ppm O_3_ for 3 h to replicate pulmonary neutrophilia observed at 400 ppb in controlled exposure human studies that leads to pulmonary neutrophilia [116]. Thus, utilized concentrations model an acute pollution episode as well as a single day occupational exposure at the current PEL.

Despite reported novel observations of inhalation co-exposure-induced gut and lung microbiome changes and systemic mediator alterations, our study does have limitations. First, while inhalation exposure changed microbial abundance and/or composition in the lung and gut and altered gut derived metabolic mediators, the mechanism of such changes and implication in metabolic disease pathogenesis still need to be addressed. Second, we only used male mice to investigate potential alterations in the microbiome and further research should include both male and female mice. Finally, ingestion of particles through grooming can potentially impact microbiome and is a potential draw back of the whole-body inhalation exposures. However, whole body inhalation exposures are known to be less stressful as these don’t require restraint.

## Conclusion

In summary, our study demonstrates that inhalation co-exposure is associated with induction of pulmonary inflammatory response as well as microbiome alterations in the lungs and gut. Interestingly, the gut and lung microbiome does not show a similar trend/extent of change, with gut microbiota revealing an active homeostatic shift potentially to counter the pulmonary adverse outcomes of inhalation exposure. Moreover, we demonstrate that inhalation co-exposure induced systemic oxidant response and induced altered intestinal epithelial cell driven mediator mRNA expression indicating a systemic impact of inhalation co-exposure to CB and O_3_. Further work should elaborate the link between inflammation and microbiome alterations in the lung microbiome as well as further examine gut-lung coordination after inhalation exposures.

## Supplementary Information

Additional file 1. Sequence data are available in NCBI database BioProject PRJNA891236. The online version contains additional file available at journal website. It includes. Figure S1: Changes in weight after single and multiple CB, O3 and CB + O3 inhalation exposures. Figure S2: Gating strategy for identifying inflammatory cells in lungs. A Flow gating strategy to detect cell types in lungs after air, CB, O3 or CB + O3 exposure either for 3 h for one day or four days. B Inflammatory cells identification markers. C Inflammatory cell markers, conjugated fluorochrome, clone and vendors. Figure S3: Exposure induce changes in bacterial community in the lungs. A Alterations in the Lactobacillus in response to inhalation exposure in the lungs. B, C Principle Co-ordinate analysis of lung microbial community, Bray–Curtis, and Unweighted Unifrac, respectively. Figure S4: Exposure induce changes in beta diversity indices in colon contents microbiome. A–C Principle Co-ordinate analysis of colon content microbial community, Bray–Curtis, Jaccard distance and Unweighted Unifrac, respectively. D Alterations in the Firmicutes to Bacteroidetes ratio in the colon content. Figure S5: Correlation analysis of absolute bacterial load in the colon contents to oxidative stress.

## Data Availability

All sequencing data and code have been made freely available. Raw and processed transcriptomic reads have been added here: https://www.ncbi.nlm.nih.gov/bioproject/PRJNA891236. Raw data values used for generation of heat maps for flow analyses and Real time PCR and primer sequences are provided in Additional File. Other data sets analyzed in this study are available from corresponding author on reasonable request.
