# Supplementary material for: Lung-gut axis of microbiome alterations following co-exposure to ultrafine carbon black and ozone
Source: Part Fibre Toxicol. 2023 Apr 21;20:15. doi: 10.1186/s12989-023-00528-8 (PMC10122302; doi:10.1186/s12989-023-00528-8)
Supplement: Supplementary file 1 — Additional file 1. Sequence data are available in NCBI database BioProject PRJNA891236. The online version contains additional file available at journal website. It includes. Figure S1: Changes in weight after single and multiple CB, O3 and CB + O3 inhalation exposures. Figure S2: Gating strategy for identifying inflammatory cells in lungs. A Flow gating strategy to detect cell types in lungs after air, CB, O3 or CB + O3 exposure either for 3 h for one day or four days. B Inflammatory cells identification markers. C Inflammatory cell markers, conjugated fluorochrome, clone and vendors. Figure S3: Exposure induce changes in bacterial community in the lungs. A Alterations in the Lactobacillus in response to inhalation exposure in the lungs. B, C Principle Co-ordinate analysis of lung microbial community, Bray–Curtis, and Unweighted Unifrac, respectively. Figure S4: Exposure induce changes in beta diversity indices in colon contents microbiome. A–C Principle Co-ordinate analysis of colon content microbial community, Bray–Curtis, Jaccard distance and Unweighted Unifrac, respectively. D Alterations in the Firmicutes to Bacteroidetes ratio in the colon content. Figure S5: Correlation analysis of absolute bacterial load in the colon contents to oxidative stress. [file 12989_2023_528_MOESM1_ESM.pptx]

## Slide 1
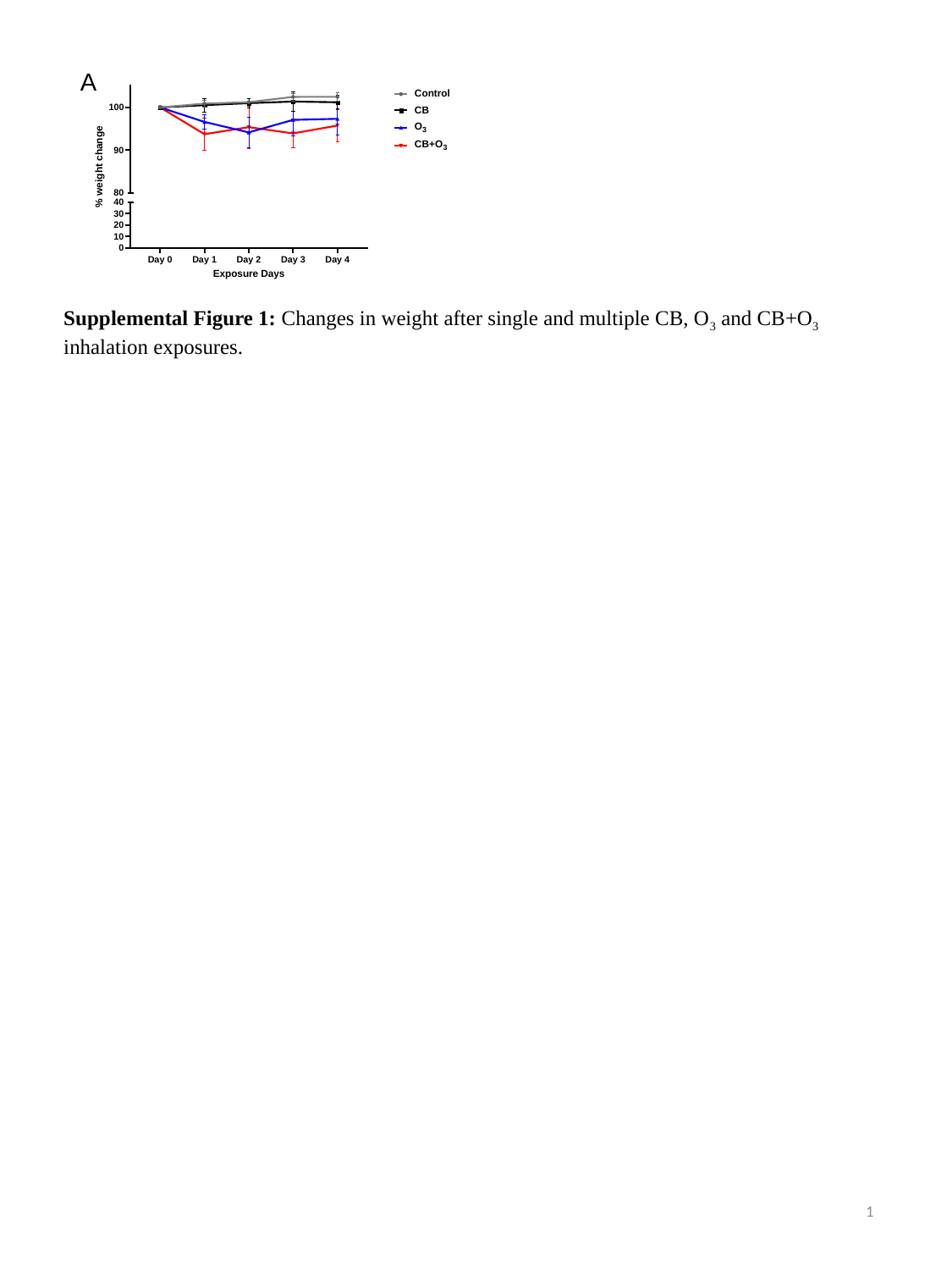

A
Supplemental Figure 1: Changes in weight after single and multiple CB, O3 and CB+O3 inhalation exposures.
1

## Slide 2
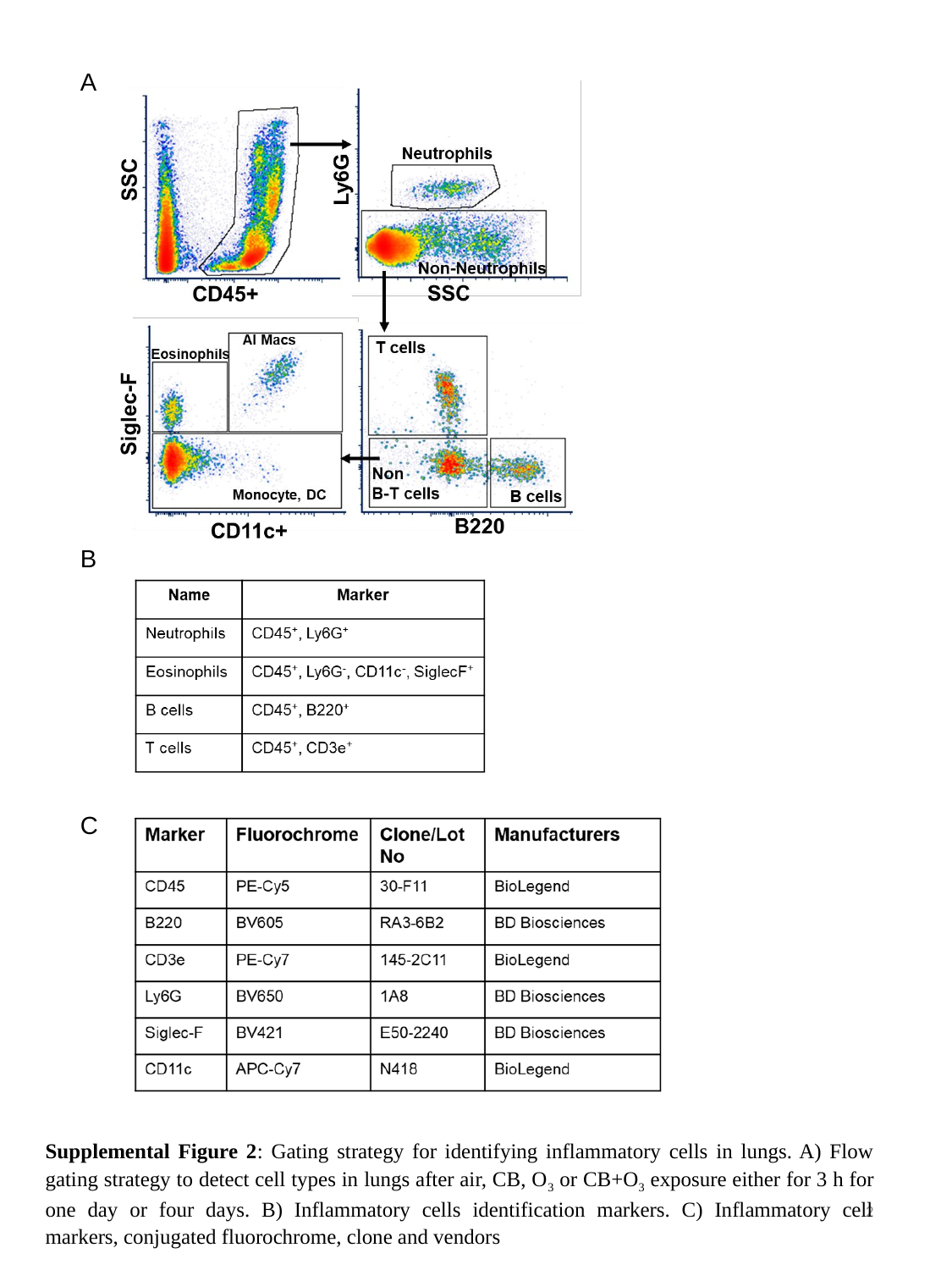

A
B
C
Supplemental Figure 2: Gating strategy for identifying inflammatory cells in lungs. A) Flow gating strategy to detect cell types in lungs after air, CB, O3 or CB+O3 exposure either for 3 h for one day or four days. B) Inflammatory cells identification markers. C) Inflammatory cell markers, conjugated fluorochrome, clone and vendors
2

## Slide 3
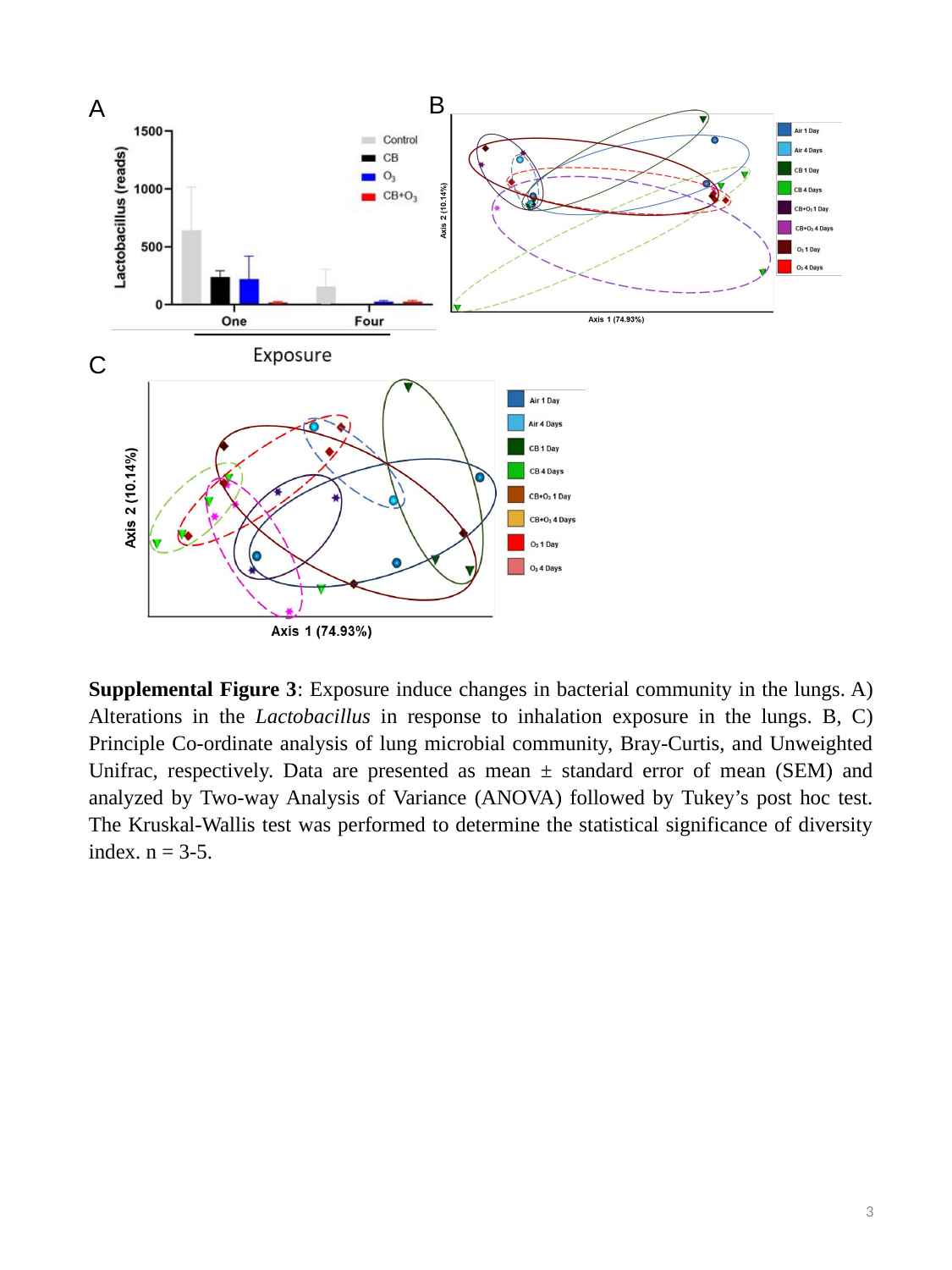

B
A
C
Supplemental Figure 3: Exposure induce changes in bacterial community in the lungs. A) Alterations in the Lactobacillus in response to inhalation exposure in the lungs. B, C) Principle Co-ordinate analysis of lung microbial community, Bray-Curtis, and Unweighted Unifrac, respectively. Data are presented as mean ± standard error of mean (SEM) and analyzed by Two-way Analysis of Variance (ANOVA) followed by Tukey’s post hoc test. The Kruskal-Wallis test was performed to determine the statistical significance of diversity index. n = 3-5.
3

## Slide 4
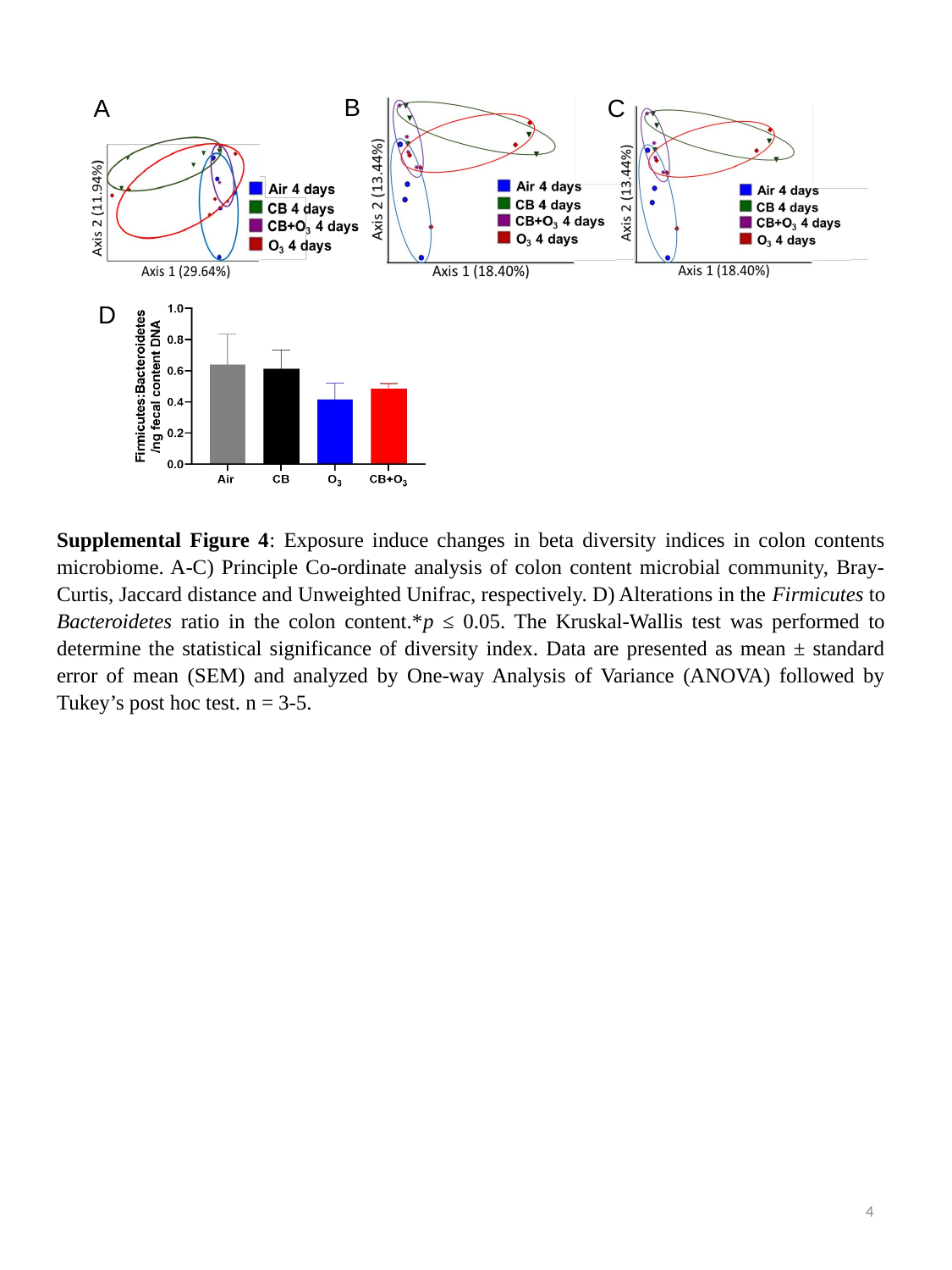

B
A
C
D
Supplemental Figure 4: Exposure induce changes in beta diversity indices in colon contents microbiome. A-C) Principle Co-ordinate analysis of colon content microbial community, Bray-Curtis, Jaccard distance and Unweighted Unifrac, respectively. D) Alterations in the Firmicutes to Bacteroidetes ratio in the colon content.*p ≤ 0.05. The Kruskal-Wallis test was performed to determine the statistical significance of diversity index. Data are presented as mean ± standard error of mean (SEM) and analyzed by One-way Analysis of Variance (ANOVA) followed by Tukey’s post hoc test. n = 3-5.
4

## Slide 5
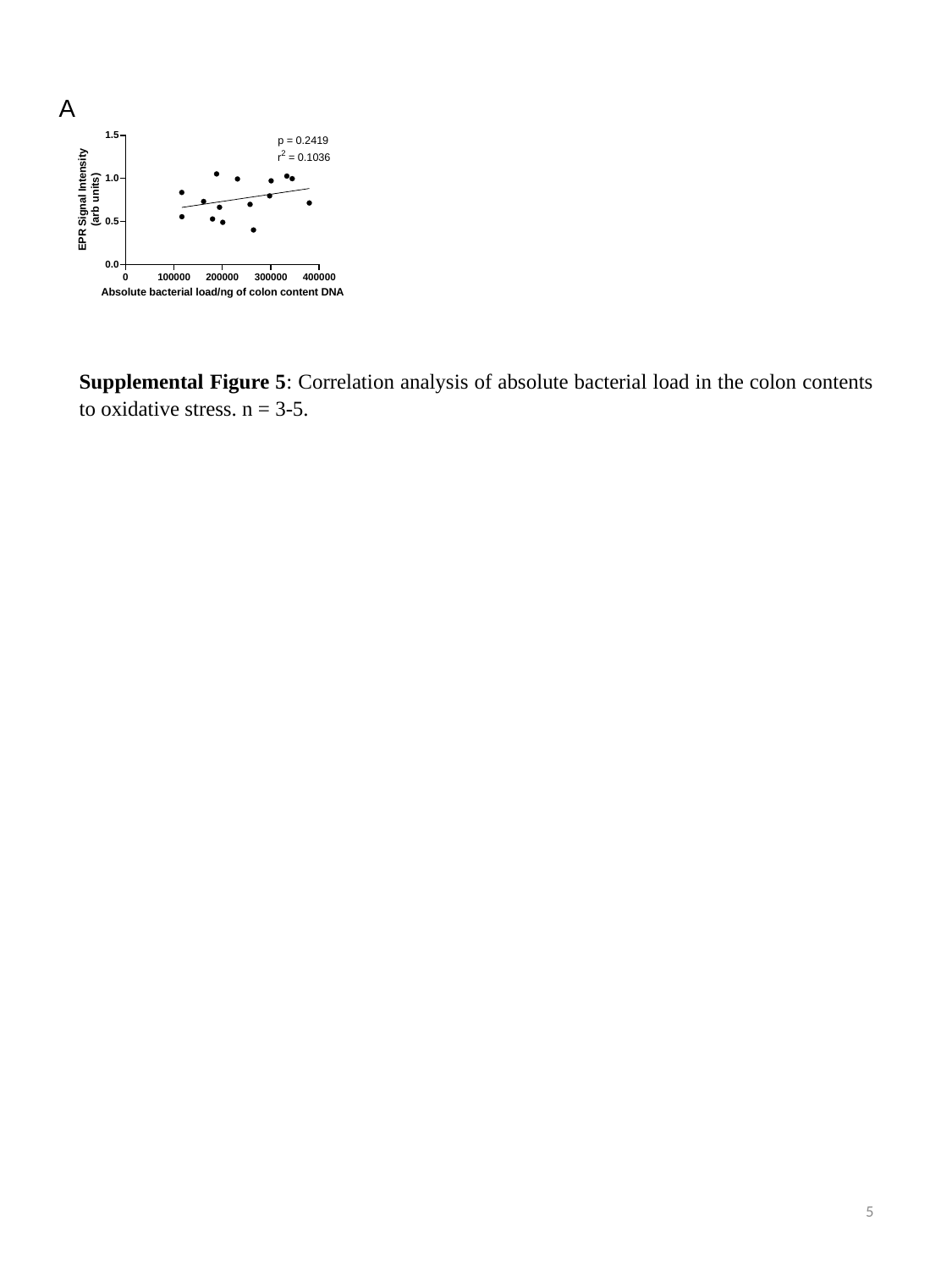

A
Supplemental Figure 5: Correlation analysis of absolute bacterial load in the colon contents to oxidative stress. n = 3-5.
5

## Slide 6
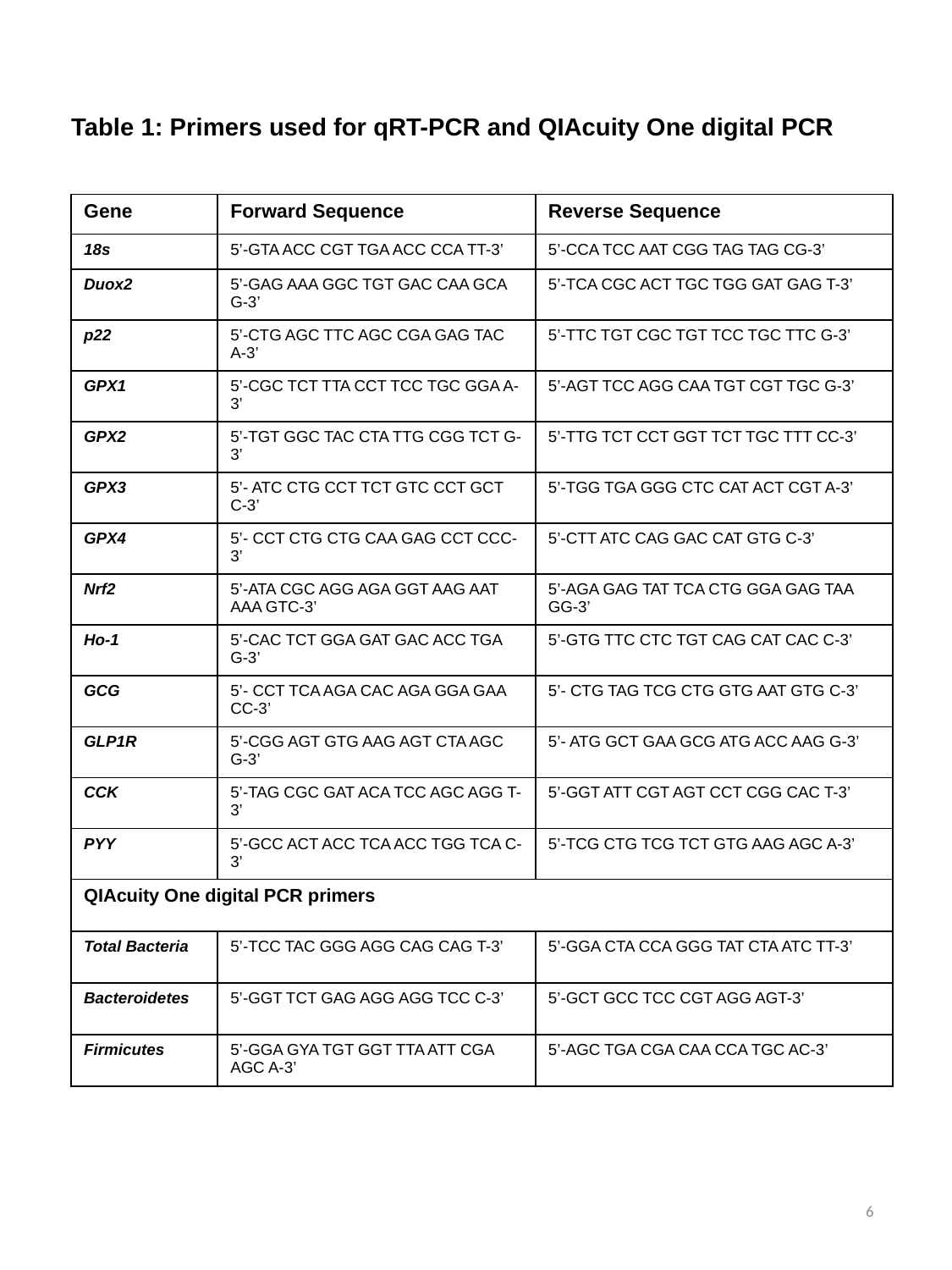

Table 1: Primers used for qRT-PCR and QIAcuity One digital PCR
| Gene | Forward Sequence | Reverse Sequence |
| --- | --- | --- |
| 18s | 5’-GTA ACC CGT TGA ACC CCA TT-3’ | 5’-CCA TCC AAT CGG TAG TAG CG-3’ |
| Duox2 | 5’-GAG AAA GGC TGT GAC CAA GCA G-3’ | 5’-TCA CGC ACT TGC TGG GAT GAG T-3’ |
| p22 | 5’-CTG AGC TTC AGC CGA GAG TAC A-3’ | 5’-TTC TGT CGC TGT TCC TGC TTC G-3’ |
| GPX1 | 5’-CGC TCT TTA CCT TCC TGC GGA A-3’ | 5’-AGT TCC AGG CAA TGT CGT TGC G-3’ |
| GPX2 | 5’-TGT GGC TAC CTA TTG CGG TCT G-3’ | 5’-TTG TCT CCT GGT TCT TGC TTT CC-3’ |
| GPX3 | 5’- ATC CTG CCT TCT GTC CCT GCT C-3’ | 5’-TGG TGA GGG CTC CAT ACT CGT A-3’ |
| GPX4 | 5’- CCT CTG CTG CAA GAG CCT CCC-3’ | 5’-CTT ATC CAG GAC CAT GTG C-3’ |
| Nrf2 | 5’-ATA CGC AGG AGA GGT AAG AAT AAA GTC-3’ | 5’-AGA GAG TAT TCA CTG GGA GAG TAA GG-3’ |
| Ho-1 | 5’-CAC TCT GGA GAT GAC ACC TGA G-3’ | 5’-GTG TTC CTC TGT CAG CAT CAC C-3’ |
| GCG | 5’- CCT TCA AGA CAC AGA GGA GAA CC-3’ | 5’- CTG TAG TCG CTG GTG AAT GTG C-3’ |
| GLP1R | 5’-CGG AGT GTG AAG AGT CTA AGC G-3’ | 5’- ATG GCT GAA GCG ATG ACC AAG G-3’ |
| CCK | 5’-TAG CGC GAT ACA TCC AGC AGG T-3’ | 5’-GGT ATT CGT AGT CCT CGG CAC T-3’ |
| PYY | 5’-GCC ACT ACC TCA ACC TGG TCA C-3’ | 5’-TCG CTG TCG TCT GTG AAG AGC A-3’ |
| QIAcuity One digital PCR primers | | |
| Total Bacteria | 5’-TCC TAC GGG AGG CAG CAG T-3’ | 5’-GGA CTA CCA GGG TAT CTA ATC TT-3’ |
| Bacteroidetes | 5’-GGT TCT GAG AGG AGG TCC C-3’ | 5’-GCT GCC TCC CGT AGG AGT-3’ |
| Firmicutes | 5’-GGA GYA TGT GGT TTA ATT CGA AGC A-3’ | 5’-AGC TGA CGA CAA CCA TGC AC-3’ |
6

## Slide 7
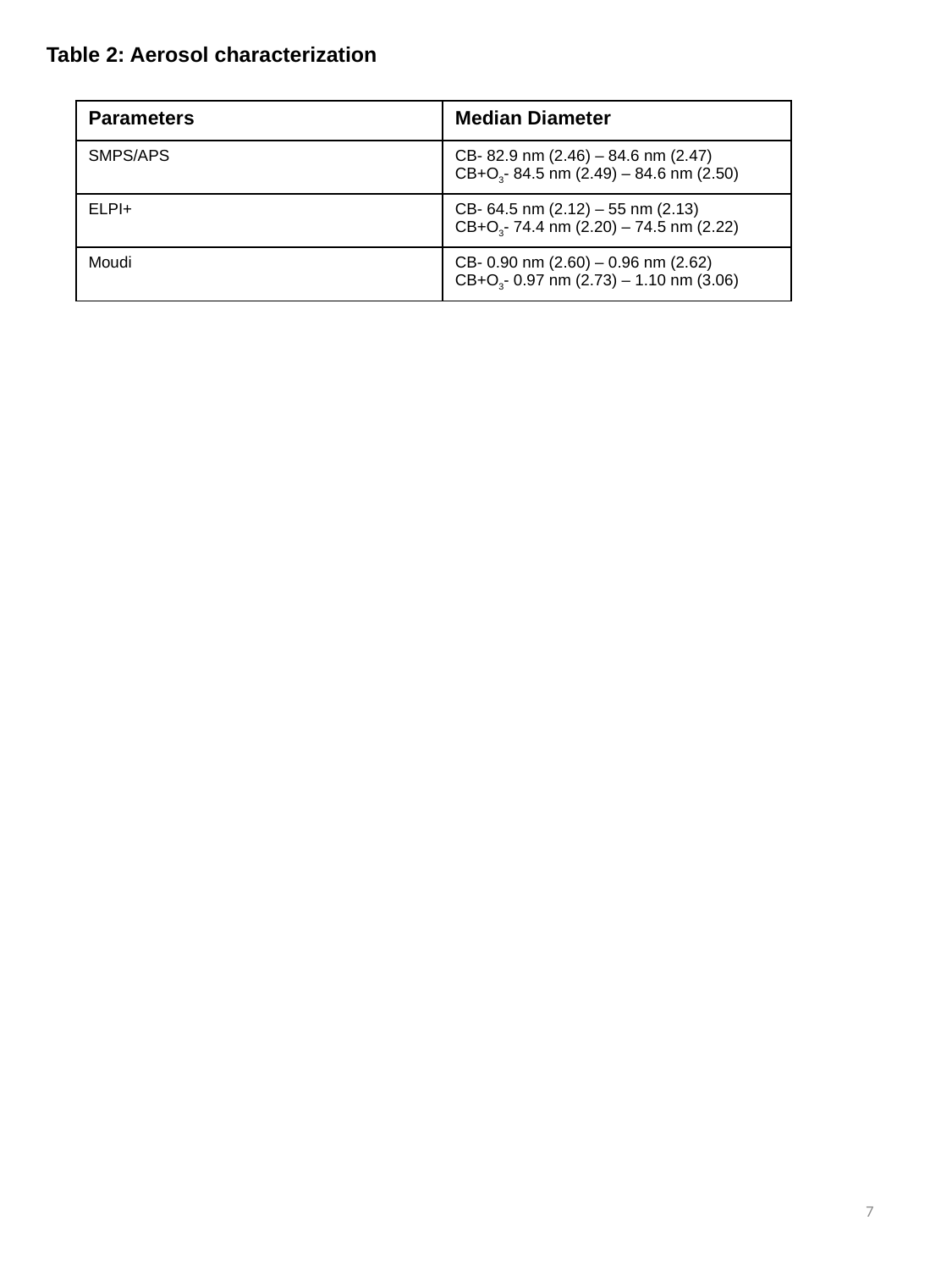

Table 2: Aerosol characterization
| Parameters | Median Diameter |
| --- | --- |
| SMPS/APS | CB- 82.9 nm (2.46) – 84.6 nm (2.47) CB+O3- 84.5 nm (2.49) – 84.6 nm (2.50) |
| ELPI+ | CB- 64.5 nm (2.12) – 55 nm (2.13) CB+O3- 74.4 nm (2.20) – 74.5 nm (2.22) |
| Moudi | CB- 0.90 nm (2.60) – 0.96 nm (2.62) CB+O3- 0.97 nm (2.73) – 1.10 nm (3.06) |
7

## Slide 8
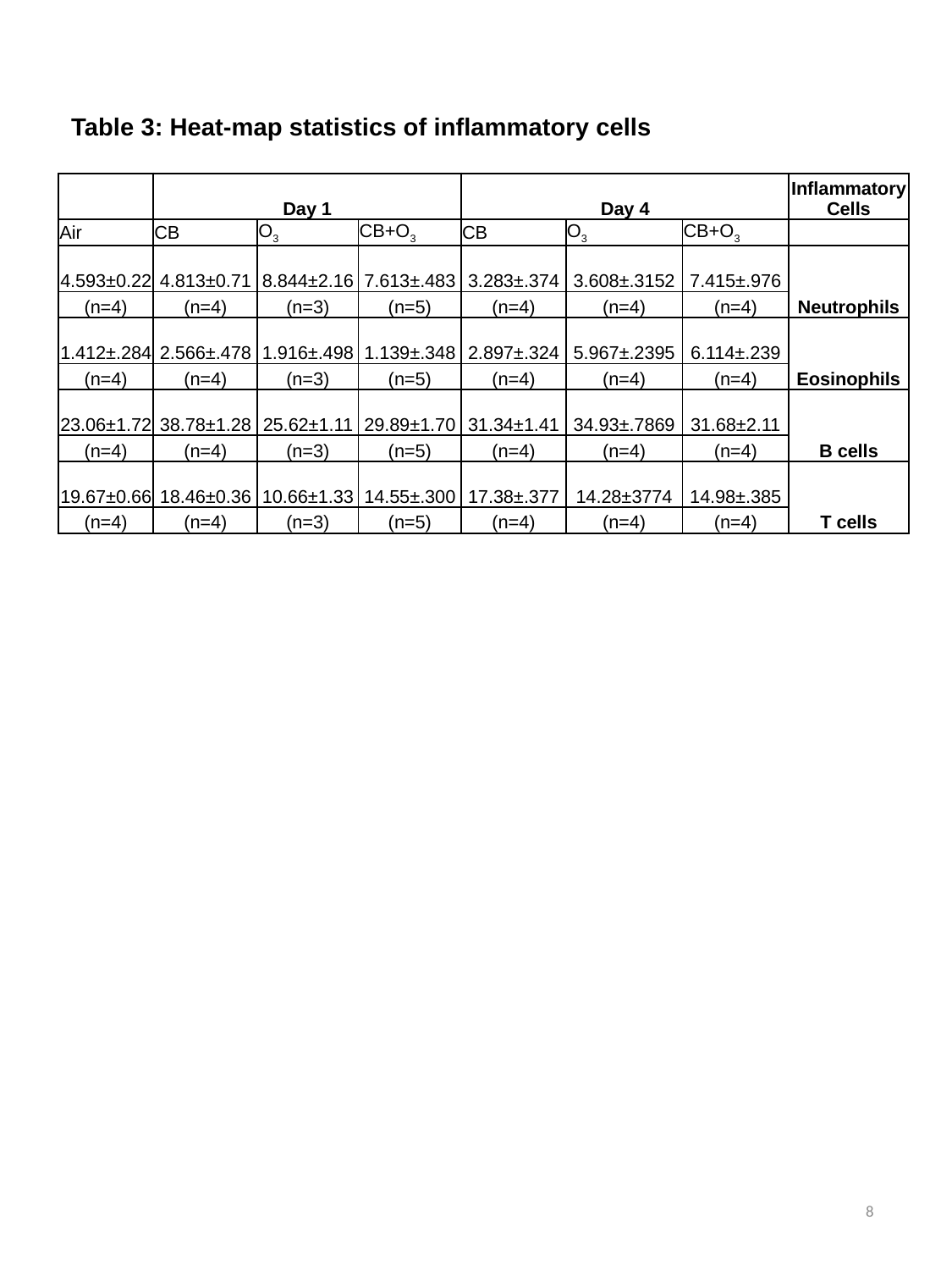

Table 3: Heat-map statistics of inflammatory cells
| | | | | | | | | | | | |
| --- | --- | --- | --- | --- | --- | --- | --- | --- | --- | --- | --- |
| | Day 1 | | | | | Day 4 | | | | | Inflammatory Cells |
| Air | CB | | O3 | | CB+O3 | CB | | O3 | | CB+O3 | |
| 4.593±0.22 | 4.813±0.71 | | 8.844±2.16 | | 7.613±.483 | 3.283±.374 | | 3.608±.3152 | | 7.415±.976 | Neutrophils |
| (n=4) | (n=4) | | (n=3) | | (n=5) | (n=4) | | (n=4) | | (n=4) | |
| 1.412±.284 | 2.566±.478 | | 1.916±.498 | | 1.139±.348 | 2.897±.324 | | 5.967±.2395 | | 6.114±.239 | Eosinophils |
| (n=4) | (n=4) | | (n=3) | | (n=5) | (n=4) | | (n=4) | | (n=4) | |
| 23.06±1.72 | 38.78±1.28 | | 25.62±1.11 | | 29.89±1.70 | 31.34±1.41 | | 34.93±.7869 | | 31.68±2.11 | B cells |
| (n=4) | (n=4) | | (n=3) | | (n=5) | (n=4) | | (n=4) | | (n=4) | |
| 19.67±0.66 | 18.46±0.36 | | 10.66±1.33 | | 14.55±.300 | 17.38±.377 | | 14.28±3774 | | 14.98±.385 | T cells |
| (n=4) | (n=4) | | (n=3) | | (n=5) | (n=4) | | (n=4) | | (n=4) | |
8

## Slide 9
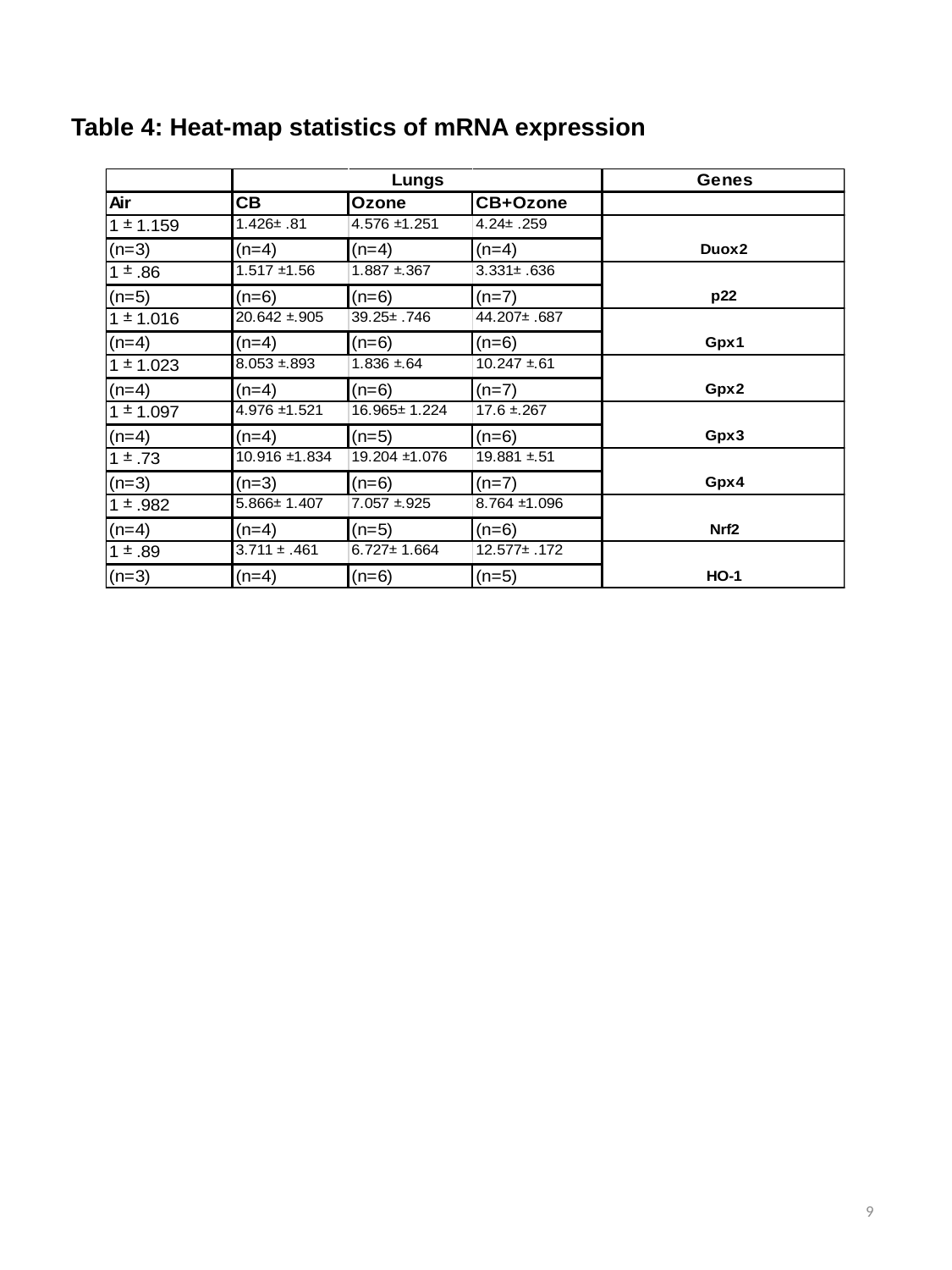

Table 4: Heat-map statistics of mRNA expression
9
